# Scrooge: a fast and memory-frugal genomic sequence aligner for CPUs, GPUs, and ASICs

**DOI:** 10.1093/bioinformatics/btad151

**Published:** 2023-03-24

**Authors:** Joël Lindegger, Damla Senol Cali, Mohammed Alser, Juan Gómez-Luna, Nika Mansouri Ghiasi, Onur Mutlu

**Affiliations:** Department of Information Technology and Electrical Engineering, ETH Zurich, Zurich 8006, Switzerland; Bionano Genomics, San Diego, CA 92121, United States; Department of Information Technology and Electrical Engineering, ETH Zurich, Zurich 8006, Switzerland; Department of Information Technology and Electrical Engineering, ETH Zurich, Zurich 8006, Switzerland; Department of Information Technology and Electrical Engineering, ETH Zurich, Zurich 8006, Switzerland; Department of Information Technology and Electrical Engineering, ETH Zurich, Zurich 8006, Switzerland

## Abstract

**Motivation:**

Pairwise sequence alignment is a very time-consuming step in common bioinformatics pipelines. Speeding up this step requires heuristics, efficient implementations, and/or hardware acceleration. A promising candidate for all of the above is the recently proposed GenASM algorithm. We identify and address three inefficiencies in the GenASM algorithm: it has a high amount of data movement, a large memory footprint, and does some unnecessary work.

**Results:**

We propose *Scrooge*, a fast and memory-frugal genomic sequence aligner. Scrooge includes three novel algorithmic improvements which reduce the data movement, memory footprint, and the number of operations in the GenASM algorithm. We provide efficient open-source implementations of the Scrooge algorithm for CPUs and GPUs, which demonstrate the significant benefits of our algorithmic improvements. For long reads, the CPU version of Scrooge achieves a 20.1×, 1.7×, and 2.1× speedup over KSW2, Edlib, and a CPU implementation of GenASM, respectively. The GPU version of Scrooge achieves a 4.0×, 80.4×, 6.8×, 12.6×, and 5.9× speedup over the CPU version of Scrooge, KSW2, Edlib, Darwin-GPU, and a GPU implementation of GenASM, respectively. We estimate an ASIC implementation of Scrooge to use 3.6× less chip area and 2.1× less power than a GenASM ASIC while maintaining the same throughput. Further, we systematically analyze the throughput and accuracy behavior of GenASM and Scrooge under various configurations. As the best configuration of Scrooge depends on the computing platform, we make several observations that can help guide future implementations of Scrooge.

**Availability and implementation:**

https://github.com/CMU-SAFARI/Scrooge.

## 1 Introduction

*Pairwise sequence alignment* is a computational step commonly required in bioinformatics pipelines (Alser et al. 2022), such as in *read mapping* (Alser et al. 2020a) and *de novo assembly* (Li et al. 2011). We formulate the problem as: (i) finding the *edit distance* between two sequences (Levenshtein 1966) and (ii) determining the sequence of corresponding edits. Efficient algorithms for solving this problem optimally are based on *dynamic programming (DP)*, such as the Smith–Waterman–Gotoh algorithm (Smith and Waterman 1981; Gotoh 1982), and have a runtime that grows quadratically with sequence length (Alser et al. 2021). Backurs and Indyk (2015) proves no strongly subquadratic time solutions can exist, provided the strong exponential time hypothesis (Impagliazzo and Paturi 2001) holds. Hence, recent works focus on approaches such as prealignment filtering *(*e.g. Xin et al. 2013, 2015; Alser et al. 2019, 2020b; Singh et al. 2021; Mansouri Ghiasi et al. 2022), constant factor algorithmic speedups *(*e.g. Šošić and Šikić 2017; Li 2018; Suzuki and Kasahara 2018; Marco-Sola et al. 2020), GPU-based acceleration *(*e.g. Liu et al. 2013; de Oliveira Sandes et al. 2016; Ahmedet al. 2019, 2020; Awan et al. 2020), FPGA-based acceleration *(*e.g. Benkrid et al. 2009; Hoffmann et al. 2016; Feiet al. 2018), or using specialized hardware accelerators *(*e.g. Fujiki et al. 2018, 2020; Turakhiaet al. 2018, 2019; Senol Cali et al. 2020, 2022).

We observe that GenASM (Senol Cali et al. 2020), a recent state-of-the-art sequence alignment algorithm, has a large space for improvement. GenASM uses only cheap bitwise operations and breaks the lower complexity bound of pairwise sequence alignment through its powerful *windowing heuristic*. Senol Cali et al. (2020) has already proven the effectiveness of the GenASM algorithm and its accelerator implementation, thus we are motivated to further improve the GenASM algorithm and explore its potential on commodity hardware.

We identify three inefficiencies in the GenASM algorithm: (i) it has a *large memory footprint* due to the large size of the dynamic programming (DP) table, (ii) it has a *high amount of data movement* between registers and memory due to frequent accesses to the DP table, and (iii) it does some *unnecessary* work by calculating DP cells that are not useful for finding the final result. The three inefficiencies negatively impact both (i) software implementations running on commodity hardware (e.g. CPUs or GPUs) and (ii) custom hardware (e.g. ASIC) implementations.

*Software implementations* on commodity hardware typically cannot fit all the data into fast on-chip memories (e.g. L1, scratchpad memory) due to the large memory footprint. This increases the latency and limits the bandwidth with which the DP table can be accessed. The high amount of data movement puts high pressure on this bandwidth, limiting performance.

In contrast, *custom hardware implementations* can use arbitrarily large amounts of on-chip memory, but such a large on-chip memory with the high bandwidth requirement is costly. For example, the hardware accelerator described in Senol Cali et al. (2020) requires 76% and 54% of the total chip area and power consumption for the on-chip memory that stores the DP table.

The unnecessary work stems from computing cells that do not contain useful information for finding the final result. This applies to at least 25% of cells on an average for uncorrelated string pairs, and more for correlated string pairs, as we show in Section 2.4.3. Doing unnecessary work affects software and hardware implementations equally because both could use the wasted time to do useful work instead.

Our goal is to develop a fast and memory-frugal alignment algorithm by addressing the inefficiencies in the GenASM algorithm, and demonstrate its benefits with high-performance CPU and GPU implementations.

To this end we propose Scrooge, which includes improvements to the GenASM algorithm based on three key ideas:

- The DP table can be *compressed* by storing only the bitwise AND of multiple values (see Section 2.4.1). The required regions of the DP table can then be decompressed on-demand during traceback with a small computational overhead.
- Part of the DP table *does not need to be stored* because the traceback operation cannot reach these entries (see Section 2.4.2).
- Part of the DP table can opportunistically be *excluded from calculation* if previous rows of the DP table already contain the information needed for finding the final result (see Section 2.4.3).

These improvements (i) reduce the number of accesses to GenASMs DP table, (ii) reduce the memory footprint of the DP table, and (iii) eliminate unnecessary work. *Scrooge* is a name for miserly or frugal fictional characters (e.g., Dickens 1843), similar to how our proposed algorithm aims to be as resource-efficient as possible.

We experimentally demonstrate that our improvements yield significant benefits across multiple computing platforms and multiple baseline sequence alignment methods. The CPU version of Scrooge achieves a 20.1×, 1.7×, and 2.1× speedup over CPU-based implementations of KSW2 (Li 2018; Suzuki and Kasahara 2018), Edlib (Šošić and Šikić 2017), GenASM, respectively. The GPU version of Scrooge achieves a 4.0×, 80.4×, 6.8×, 12.6×, and 5.9× speedup over CPU-based implementations of Scrooge, KSW2, and Edlib, and GPU-based implementations of Darwin-GPU (Ahmed et al. 2020) and GenASM, respectively. We analytically estimate an ASIC implementation of Scrooge to use 3.6× less chip area and consume 2.1× less power compared to the prior state-of-the-art ASIC implementation of GenASM (Senol Cali et al. 2020) while maintaining the same throughput.

The contributions of this paper are as follows:

- We develop three novel algorithmic improvements that are applicable to software and custom hardware implementations of Scrooge, collectively reducing the memory footprint by 24×, the number of memory accesses by 12×, and the number of entries of the DP table calculated by at least 25% on an average compared to GenASM.
- We experimentally demonstrate the significant throughput (i.e. alignments per second) increase of our improvements for CPU and GPU implementations of Scrooge.
- We analytically estimate that an ASIC implementation of Scrooge significantly reduces the chip area and power consumption compared to the prior state-of-the-art ASIC implementation of GenASM.
- We open-source all code, including high-performance CPU and GPU implementations of Scrooge, which can be readily used as a sequence alignment library, and all evaluation scripts.
- We systematically analyze the throughput and accuracy behavior of GenASM and Scrooge across a range of configurations based on real and simulated datasets for long and short reads. As the best configuration of Scrooge depends on the computing platform, we make several observations that can help guide future implementations of Scrooge.

## 2 Materials and methods

### 2.1 Overview

The primary purpose of Scrooge is to accelerate pairwise sequence alignment through (i) a memory-frugal and efficient algorithm, and (ii) optimized CPU, GPU, and ASIC implementations.

Scrooge solves the *approximate string matching (ASM)* problem with the *edit distance* (Levenshtein 1966) as the cost metric. That is, given two strings, text and pattern, Scrooge finds the minimum number of single-letter substitutions, insertions, and deletions to convert text into pattern. Additionally, the sequence of edits that corresponds the edit distance is reported, which is called *CIGAR string*.

The Scrooge algorithm is based on the GenASM algorithm (see Section 2.2). Senol Cali et al. (2020) first proposed the GenASM algorithm as an algorithm/hardware co-design targeted for an ASIC accelerator, and demonstrated GenASMs potential for very high throughput and resource efficiency. However, as we show in Section 2.3, the GenASM algorithm: (i) requires large amounts of memory bandwidth, (ii) exhibits a large memory footprint, and (iii) does some unnecessary work. These inefficiencies limit GenASMs throughput and resource efficiency on both commodity and custom hardware, and addressing them is critical.

To this end, we propose Scrooge’s three novel algorithmic improvements to GenASM in Section 2.4. In Section 3.2, we experimentally demonstrate that these improvements significantly increase performance on recent CPUs and GPUs. In Section 3.4, we explore the throughput behavior of GenASM with and without the proposed improvements across various configurations. We show in Section 3.5 that an ASIC implementation of Scrooge will have significantly reduced chip area and power consumption compared to the ASIC designed for GenASM (Senol Cali et al. 2020) while maintaining the same throughput. In Section 3.6, we explore the accuracy behavior of GenASM and Scrooge across various configurations.

### 2.2 GenASM algorithm

The GenASM algorithm (Senol Cali et al. 2020) consists of two subalgorithms: *GenASM-DC* and *GenASM-TB*. GenASM-DC (see Section 2.2.1) fills a bitvector-based dynamic programming table. The last column of the table indicates the edit distance between the two input strings. GenASM-TB (see Section 2.2.2) re-traces this optimal solution in the constructed table. To better scale with longer input sequences, GenASM uses a *windowing heuristic* (see Section 2.2.3).

#### 2.2.1 GenASM-DC algorithm

**Figure 1.**
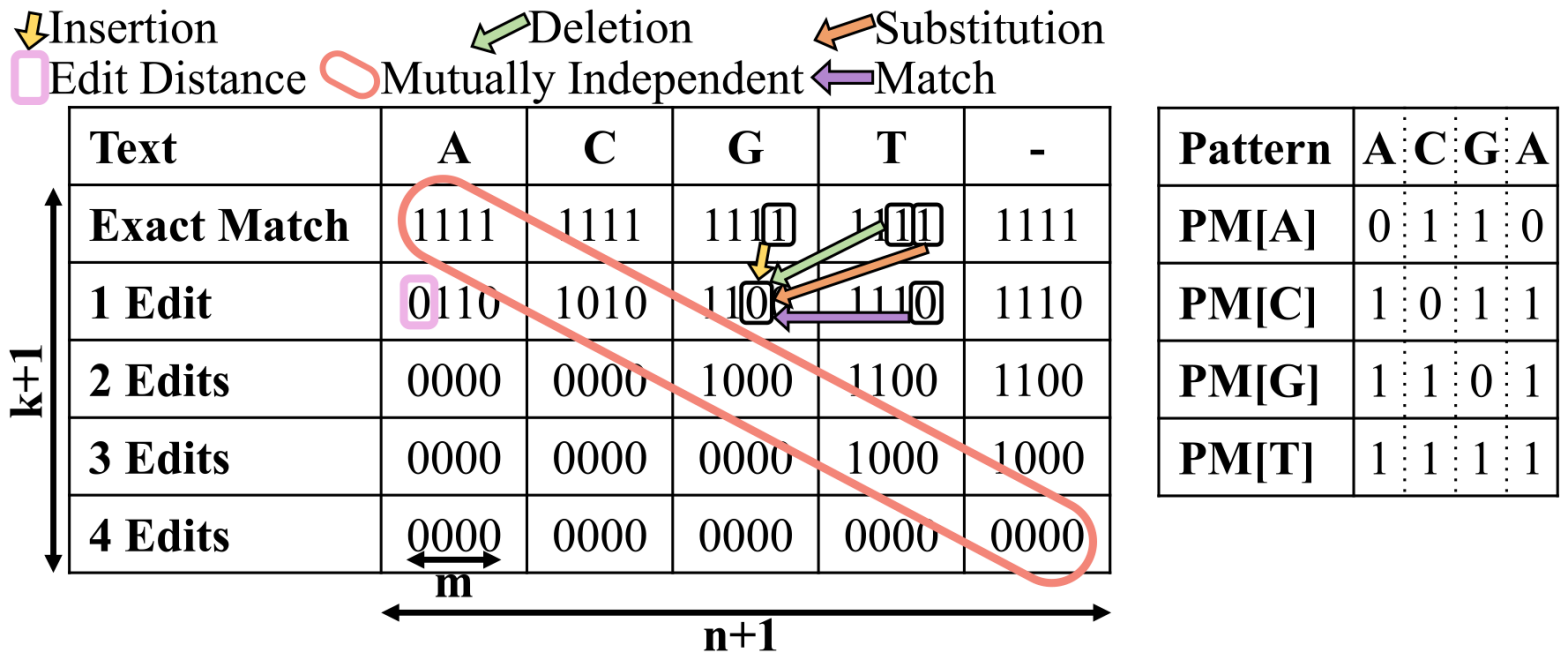
An example of DP table R with text=ACGT and k = 4. The bitmasks for pattern=ACGA are shown on the right. The colored arrows show the possible origins and data dependencies of the 0 at d = 1, i = 2, j = 2. The values in the red marked diagonal are mutually independent and thus can be computed in parallel.

GenASM-DC uses only cheap bitwise operations to calculate the edit distance between two strings text and pattern (Senol Cali et al. 2020). It builds an (n** **+** **1)×(k** **+** **1) dynamic programming (DP) table R, where n=*length*(text) and k is the maximum number of edits considered. The entries of R are m-bit bitvectors, where m=*length*(pattern). Figure 1 shows an example of R after it is constructed by GenASM-DC.

Theorem 1. *The entries (bitvectors) of R can be interpreted as follows:*

$$\begin{array}{c} j-\text{th bit of}\;R[i][d]=0\Leftrightarrow \\ d\mathrm{istance}(\mathrm{text}[i:n),\mathrm{pattern}[j:m))\leq d \end{array}$$

In natural language, Theorem 1 states that the jth bit of the bitvector R[i][d] is 0 exactly if the suffix of text starting at character i and the suffix of pattern starting at character j differ by at most d edits. Following this interpretation, the first row d** **=** **d${}_{\mathrm{OPT}}$ that has a 0 in the first bit (j** **=** **0) of the leftmost column (i** **=** **0) indicates that the edit distance between text and pattern is d${}_{\mathrm{OPT}}$. This bit is marked in pink in Fig. 1.

GenASM-DC (Algorithm 1) starts by preprocessing pattern into four *pattern masks*, one per character in the alphabet. The pattern mask for character X∈{A,C,G,T} is a bitvector of length m=*length*(pattern), with a 0 in the ith bit if pattern[i]==X. See Fig. 1 for an example.

GenASM-DC populates the rightmost column (Line 5) and topmost row (Line 11) of R. The remaining entries are then calculated from their respective neighbors in the north (Line 13, insertion), north-east (Lines 14–15, deletion and substitution), and east (Line 16, match) through simple bitwise update rules. We refer to (Baeza-Yates and Gonnet 1992; Wu and Manber 1992; Senol Cali et al. 2020) for detailed arguments on the correctness of GenASM-DC. To follow the rest of this paper, it is sufficient to consider: (i) the interpretation of R given in Theorem 1, and (ii) the north-east data dependencies imposed by Algorithm 1 and shown in Fig. 1.

Algorithm 1. GenASM-DC Algorithm

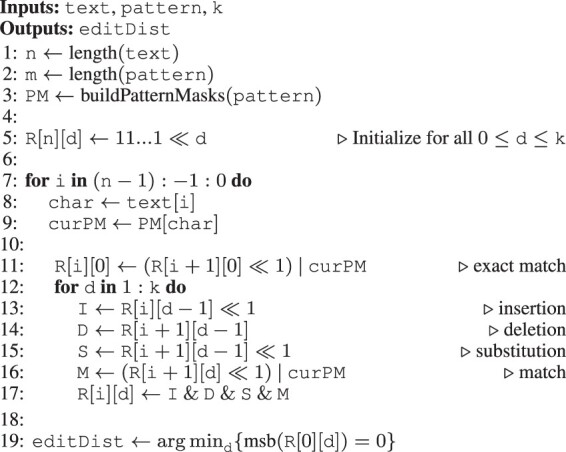

**Intra-Task Parallelism.**Senol Cali et al. (2020) enables efficient intra-task parallelism by identifying that the DP entries within each north-west to south-east diagonal (one such diagonal is marked in red in Fig. 1) do *not* depend on each other, hence they can be computed in parallel.

#### 2.2.2 GenASM-TB algorithm

For use-cases like read mapping, the pairwise sequence alignment algorithm should report both the edit distance and the corresponding sequence of edits, which is called the *CIGAR string*. Obtaining the CIGAR string involves retracing the origin of the edit distance value as a linear path through DP entries in their reverse construction order; this process is called *traceback*.

GenASM enables efficient traceback operations based on two key observations: First, if *all* intermediate values of variables I, D, S, and M in Algorithm 1 are stored, then one can follow the path of 0 s in these variables, starting from 0 in the west of R that indicates the edit distance (highlighted in pink in Fig. 1) and go towards the north-east corner of R. Whenever a 0 in one of these variables is traversed, the name of that variable is recorded as an edit (e.g. ’I’ for an insertion). Second, it is sufficient to store only three out of the four variables (because S can be obtained by shifting D), saving both memory footprint and bandwidth.

#### 2.2.3 GenASMs windowing heuristic

To provide a linear runtime complexity, Senol Cali et al. (2020) proposes a greedy *windowing heuristic*. Instead of aligning text and pattern in a single run of GenASM-DC, the windowing heuristic runs GenASM-DC multiple times as a subroutine in *windows* of size W. In each window, a prefix of size W characters of each sequence (i.e. text[0:W) and pattern[0:W)) are aligned. The first W−O characters of the window are greedily considered aligned optimally, where we call O the window *overlap*. The smaller strings text[W−O:n) and pattern[W−O:m) then remain to be aligned in the next window.

This approach has three advantages. First, instead of constructing a large table of n×m×k bits, only $\frac{m}{W-O}$ tables of $W^{3}$ bits must be constructed, saving memory footprint, data movement, and computation. Second, the GenASM-DC subroutine now runs over constant-sized sequences, simplifying its implementation. For example, DP entries can be statically assigned to processing elements (Senol Cali *et al.* 2020), and the data movement and exact memory footprint are known at compile time, even if the length of the input sequences is unknown. Third, the program flow (e.g. the number of loop iterations per window) is entirely known at compile time, giving the compiler the ability to optimize.

The windowing strategy is greedy and heuristic, so it is possible that it could miss the optimal alignment and produce a suboptimal one instead. This is a key limitation of GenASM and Scrooge. Note that several state-of-the-art tools do not give any optimality guarantees either, and instead experimentally demonstrate their practical accuracy, as Scrooge does. This includes greedy alignment techniques like SeGraM (Senol Cali et al. 2022), Darwin (Turakhia et al. 2018), and WFA-adaptive (Marco-Sola et al. 2020), as well as mappers based on sparse dynamic programming, like minimap2 (Li 2018). To balance performance and accuracy, the tunable parameters W (window size) and O (window overlap) must be selected appropriately. The parameter W can be understood as the *range* of solutions considered, similar to the *band width* (Ukkonen 1985) in popular alignment implementations (e.g. Šošić and Šikić 2017; Li 2018; Suzuki and Kasahara 2018). The parameter O can be understood as the *globality* of the solutions or *inverse greediness*. We demonstrate in Section 3: (i) that higher W and O generally improve accuracy, at the cost of lowering throughput, (ii) that the best choice of W and O depends on the input dataset (e.g. its error distribution and read lengths), and (iii) that W = 64 and O = 33 achieve a good throughput/accuracy tradeoff for long and short read mapping.

### 2.3 Inefficiencies in the GenASM algorithm

We identify three inefficiencies in the GenASM algorithm: (i) it has a large amount of data movement, (ii) it has a large memory footprint, and (iii) it does some unnecessary work.

The combination of large amount of data movement and large memory footprint, which we quantify in Sections 2.3.1 and 2.3.2, respectively, affects both software implementations running on commodity hardware, as well as custom hardware implementations. Commodity hardware (e.g. CPUs or GPUs) has a fixed amount of on-chip memory. The DP table might not fit into this on-chip memory, which introduces three inefficiencies: Data have to be moved a larger distance, which increases (i) access latency and (ii) access energy (Boroumand et al. 2018). (iii) The high amount of data movement puts high pressure on memory bandwidth, which is scarce when accessing data residing off-chip. This causes the entire application to become memory bandwidth-bound, thus wasting compute resources and achieving suboptimal performance. Custom hardware implementations (e.g. ASICs) can have as large on-chip memory as needed, but such a large and high-bandwidth on-chip memory comes at the cost of a large chip area and power consumption (Boroumand et al. 2021).

Doing unnecessary work trivially wastes runtime and energy. In Section 2.4.3, we identify the DP entries that are calculated needlessly by GenASM, and quantify how frequent they are.

#### 2.3.1 Roofline model

We use the *roofline model* (Williams et al. 2009; Ofenbeck et al. 2014) to visualize that GenASM has a large amount of data movement, and that its operational intensity (i.e. the number of operations per byte) is too low to saturate the compute resources of modern CPUs and GPUs. The roofline model plots the upper limit of achievable compute throughput for different operational intensities for a given processor. It consists of horizontal peak compute throughput rooflines, and sloped memory bandwidth rooflines.

**Figure 2.**
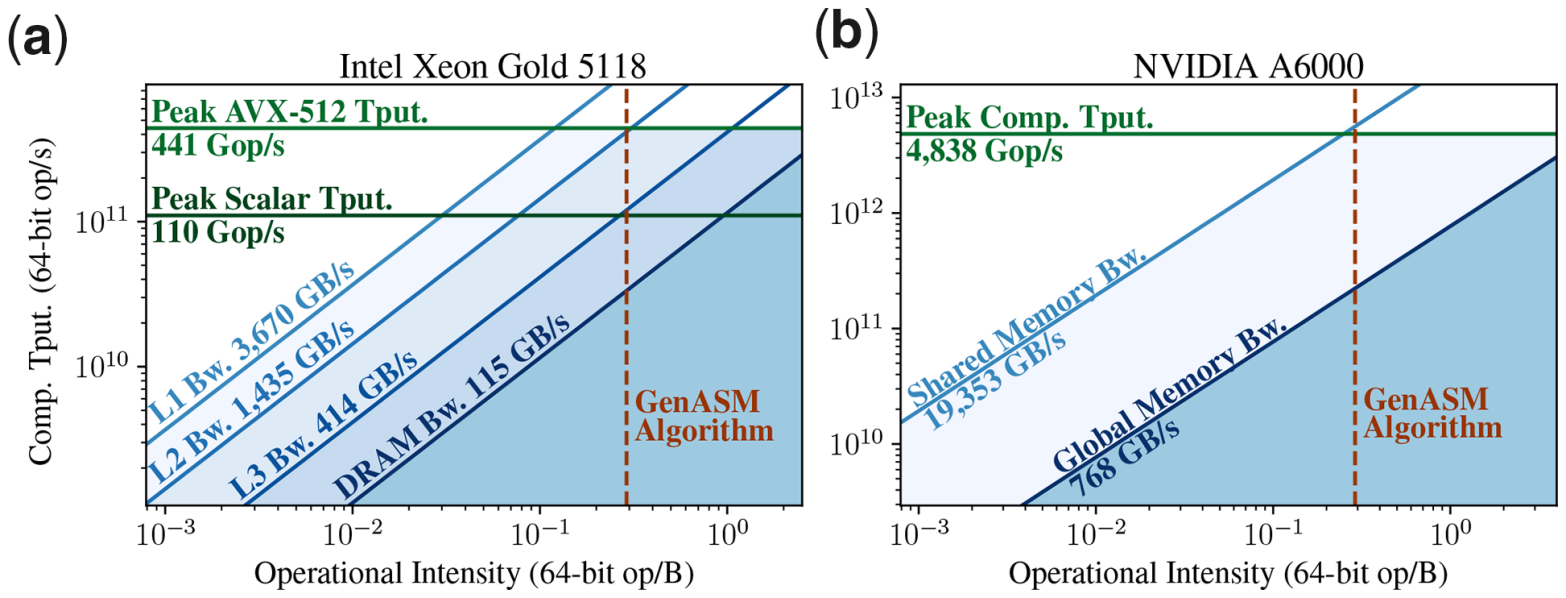
The roofline models of (a) an Intel Xeon Gold 5118 CPU and (b) an NVIDIA A6000 GPU.

Figure 2 shows the roofline plots for an Intel Xeon Gold 5118 CPU (Intel 2017) and an NVIDIA A6000 GPU (NVIDIA 2020), including their respective on-chip memory (*shared memory* in CUDA), cache, and off-chip memory (*global memory* in CUDA) bandwidths (drawn in shades of blue) and peak compute throughputs (draw in shades of green). GenASMs operational intensity is drawn in red. We derive the roofline parameters in Section 8 of the Supplementary Materials.

From Fig. 2, we make three observations. First, if the data resides off-chip, GenASM is heavily memory bandwidth-bound for a modern CPU and GPU. This is evidenced by the red (algorithm) and dark blue (off-chip memory bandwidth) lines intersecting far below the green (peak compute throughput) line. Second, GenASM would no longer be memory bandwidth-bound if its computational intensity were ≥10× higher, because then the red (algorithm) line would be shifted to the right and intersect with the dark blue (off-chip memory bandwidth) line above the green (peak compute throughput) line. The operational intensity could be increased by reducing GenASMs data movement. Third, if the data reside in the fastest on-chip memory, GenASM *can* reach peak compute throughput, even with the high amount of data movement in the baseline algorithm. This is evidenced by the red (algorithm) and light blue (L1/shared memory bandwidth) lines intersecting above the green (peak compute throughput) line. However, as we show in Section 2.3.2, GenASMs memory footprint is too large for the typical capacity of such fast on-chip memories in commodity hardware, and building large enough on-chip memories is costly.

Based on these observations, we conclude that: (i) GenASM cannot saturate commodity hardware with computation, and (ii) data movement should be reduced to address this inefficiency.

#### 2.3.2 Memory footprint

In this section, we demonstrate the overheads associated with GenASMs large memory footprint.

We derive GenASMs working set memory footprint to be 96.5*KiB* in Section 9 of the Supplementary Materials. For comparison, the Intel Xeon Gold 5118 has 32*KiB* of L1D cache per core (Intel 2017) and NVIDIAs *Ampere* GPU microarchitecture provides up to 99*KiB* of high-bandwidth on-chip memory per GPU core (*streaming multiprocessor*, *SM* in CUDA) (NVIDIA 2023). Thus, one SM can hold the DP table for exactly one GenASM problem instance in its on-chip memory. One thread block of two warps (i.e. 2×32 threads) can work on a single GenASM problem instance, but this does not saturate the compute resources in the SM. This is because modern GPUs are designed to alternate between executing *multiple* independent instruction streams for the purpose of hiding the latency of instructions (Lindholm et al. 2008). Underutilization of the compute resources in an SM due to too few independent instruction streams is called *low occupancy* and causes the unused computational resources to be wasted (NVIDIA 2023). Hence, the occupancy should be increased by working on multiple problem instances per SM. Multiple problem instances can fit into memory by *either* reducing the memory footprint per problem instance, *or* placing the DP tables into the GPUs off-chip memory, which has a much larger capacity. Our goal is the former, as we show in Section 2.3.1 that the latter is *not* an efficient a solution due to the off-chip memory’s limited bandwidth.

Custom hardware implementations (e.g. ASICs) can potentially have as large on-chip memory as needed. For example, the GenASM ASIC (Senol Cali *et al.* 2020) uses scratchpads of 96.5 *KiB* each to hold the DP tables. However, these scratchpads occupy 76% of the total chip area and consume over 54% of the chip power. This limits the performance achievable with a given chip area and power budget.

In summary, GenASM has a large memory footprint compared to typical on-chip memory capacities in commodity hardware, and while sufficiently large on-chip scratchpads can be designed for custom hardware implementations, it is costly to do so.

### 2.4 Scrooge

We have shown in Section 2.3 that GenASM has a high amount of data movement *and* high memory footprint per problem instance. We have elaborated that this combination either limits performance (on commodity hardware), or requires expensive large on-chip memories (on custom hardware), both of which are undesirable. Thus, our strategy is to reduce the GenASM algorithm’s memory footprint as much as possible while introducing minimal computational overhead. We present three novel algorithmic improvements that collectively achieve a 24× reduction in memory footprint, as well as a 12× reduction in data movement from the memory that holds the DP table.

#### 2.4.1 Improvement 1—store entries, not edges

**Figure 3.**
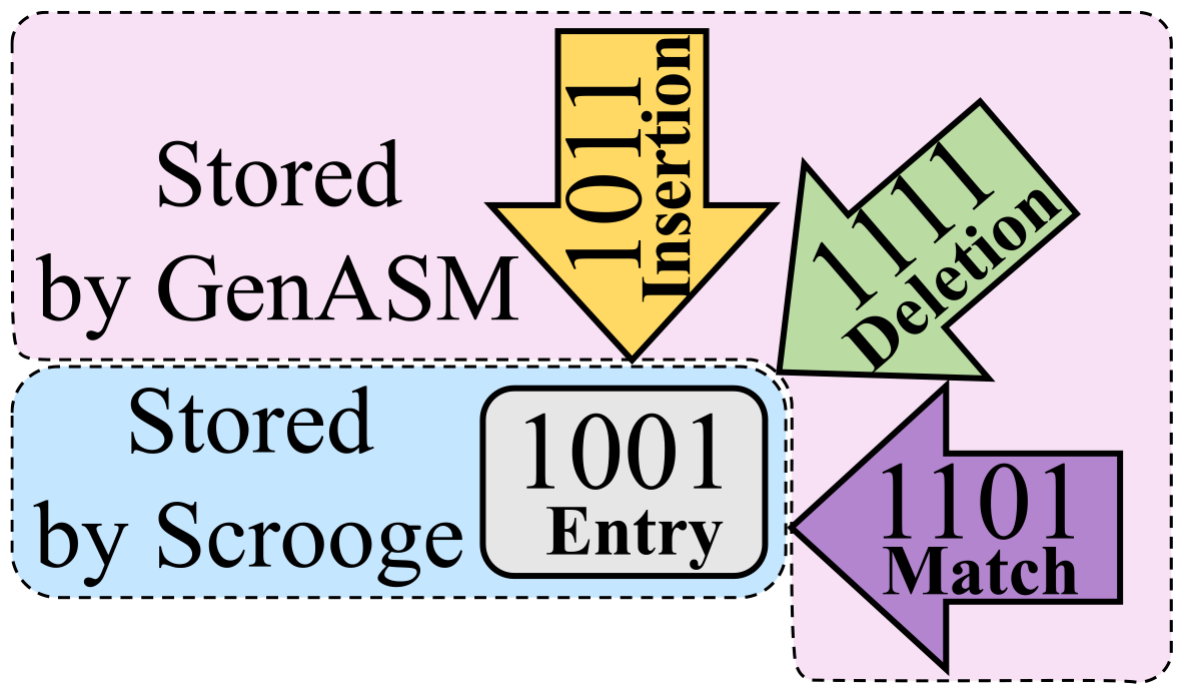
Per cell, GenASM stores three edges for traceback. Scrooge with SENE stores only the DP entry itself instead; the needed edges are regenerated on the fly during traceback.

As we explain in Section 2.2.2, GenASM stores 3 bitvectors per entry of the table R to enable traceback. If we imagine a graph where the entries of R are nodes and the intermediate bitvectors are edges connecting their source and target entries, GenASM stores 3 ingoing edges for most nodes (Fig. 3). We propose to trade off the majority of this memory footprint for a small increase in computation with the store entries, not edges (*SENE*) improvement. SENE regenerates the required edges on-demand during traceback from stored nodes (entries of table R) by applying the update rules in Algorithm 1 on requested neighbor entries.

**Cost and benefits.** Since traceback explores only a single path across the table R, only O(W) edges are regenerated, making the overhead of this extra computation small compared to computing the table of $O(W^{2})$ entries. Storing R requires storing 65×65 entries of 64 bits each, for a total of 33,800B≈33kiB. The previous memory footprint was 96.5*kiB* as derived in Section 2.3.2, yielding a $\frac{96.5}{33}=2.92\approx 3\times$ improvement in memory footprint. Since each of these locations is still only written to once during the construction of R, SENE also reduces the data movement from the memory that holds the DP table by 3×.

#### 2.4.2 Improvement 2—discard entries not used by traceback

The windowing heuristic (see Section 2.2.3) mandates that traceback covers only the first W−O characters of each window. This means that traceback never reads the table entries of the last O characters in each window.

**Figure 4.**
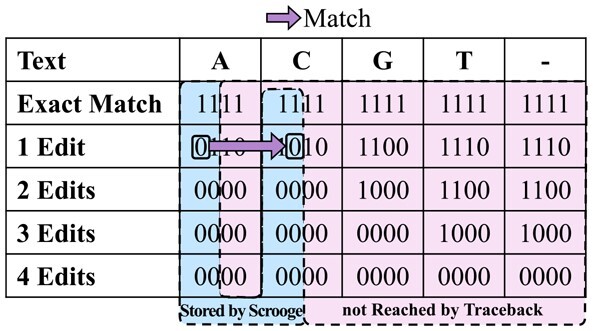
DENT exploits that the windowing heuristic stops traceback after the first W−O edges are crossed (here W = 4 and O = 3). The area never reached by traceback can be discarded.

We propose to discard the entries that can never be reached by traceback, an improvement we call discard entries not used by traceback (*DENT*). These include the last O columns of R and the last O−1 bits of every bitvector. The resulting DP table consists of W−O+1 columns, W+1 rows, and W−O+1 bits per entry. Figure 4 shows an example for W = 4 and O = 3, where Scrooge stores only the leftmost two columns and leftmost 2 bits per entry, because traceback does not reach the rightmost three columns and rightmost 2 bits per entry.

Due to the fixed word sizes and word alignment requirements of commodity hardware, the number of bits stored for each bitvector cannot be chosen freely. We show in Section 3.2 that for a modern GPU O=33 achieves the best throughput results for W=64, because the stored bitvectors perfectly fit into a 32-bit word. In contrast, Senol Cali et al. (2020) use O = 24 for its ASIC design, which we show to be suboptimal on commodity hardware. Note that increasing O*improves* accuracy, see Section 2.2.3 for an intuition and Section 3.6 for experimental results.

**Cost and benefits.** DENT incurs two computational overheads: First, the bits to store have to be determined and extracted from the bitvectors. Second, increasing O from 24 to 33 means the algorithm makes nine characters less progress per window.

By discarding the right half of each bitvector and the rightmost O columns of R, DENT improves the memory footprint by $\frac{W}{W-O+1}\times \frac{W+1}{W-O+1}=\frac{64}{32}\times \frac{65}{32}\approx 4\times$. We describe in Section 1 of the Supplementary Materials how DENT can be extended to store only half the rows of R for a total 8× memory footprint reduction.

By the same calculation, the number of writes to table R is reduced by approximately 4×, assuming the forefront diagonal (marked red in Fig. 1) is kept in registers and communicated directly.

#### 2.4.3 Improvement 3—early termination

**Figure 5.**
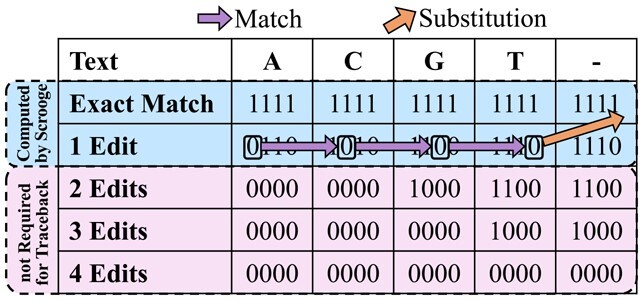
The colored edges indicate the path taken by traceback for W = 4 and O = 0. Rows below the edit distance do not contain useful information for traceback. Thus, they do not need to be computed (Early Termination).

The edit distance is determined by the highest row of R that contains a 0 in the most significant bit in the leftmost column. Traceback starts from this entry. Since entries are constructed from their north, north-east, and east neighbors, the traceback path can only go to the north, north-east, and east. It can *never* go south. Thus, at no point do the rows of higher cost than distance(pattern,text) contain useful information for traceback (see Fig. 5 for an example). We propose building R row-wise, and terminating the algorithm early as soon as the most significant bit in the first entry of the current row is a 0.

**Cost and benefits.** Early termination (ET) does not yield a constant factor improvement in either memory footprint or runtime: If distance(pattern,text)=W, we are not able to terminate early at all. However, typical input pairs incur fewer than W edits in a single window. For correct candidate pairs, the edit distance will be low, e.g. up to 15% for long reads (Alser et al. 2021). Even uncorrelated random sequence pairs of length W over a 4-letter alphabet have an edit distance of at most $\frac{3}{4}$W on an average, as we prove in Section 10 of the Supplementary Materials. Thus, on an average, Scrooge can skip at least 25% of the entries of R, saving computation as well as data movement (see Section 2.3.1).

**Conflict with intra-task parallelism.** Recall from Section 1 that GenASM provides the option for intra-task parallelism. Exploiting this parallelism requires the available processing elements to build R in a *diagonal-wise* fashion, as shown in Fig. 1. However, as we describe in Section 2.4.3, to make full use of ET, R should be built *row-wise*. As a compromise, we implement ET in a diagonal-wise fashion in our GPU implementation. As in the row-wise version, construction on R stops as soon as the leftmost processing element finds a 0 in the most significant bit. Due to the diagonal-wise computation, the other processing elements have already computed several rows ahead at this point, i.e. done unnecessary work. For this reason, the benefit of ET is limited in intra-task parallel implementations, such as our GPU implementation, while being much more significant in row-wise implementations, such as our CPU implementation. We reaffirm these effects experimentally in Section 3.4.

### 2.5 Implementation

We implement C++ versions of our algorithm for x86 CPUs and NVIDIA GPUs. They are exposed as simple library functions for pairwise sequence alignment. Each improvement and implementation constant can be easily configured at compile time through preprocessor macros. The implementations, as well as baselines and evaluation scripts, are available at https://github.com/cmu-safari/Scrooge.

**CPU.** The CPU version converts the input pairs to a two-bit-per-basepair encoding, but padded to 8 bits. Each thread works on a single pairwise alignment at a time and obtains sequence pairs from a global queue. During each call to the GenASM-DC subroutine, the thread calculates the DP table R in a row-wise fashion.

**GPU.** The GPU version is implemented using CUDA 11.1 (NVIDIA 2023) and targets GPUs of compute capability 7.0 and higher (https://developer.nvidia.com/cuda-gpus). The input sequence pairs are converted to a two-bit-per-basepair encoding and transferred to the GPU. Each thread block works on a single pairwise alignment at a time and obtains sequence pairs from a global queue. During each call to the GenASM-DC subroutine, the thread block calculates the DP table R in a diagonal-wise fashion, and each of the W threads in the thread block calculates a single column of R. Threads resolve their mutual data dependencies using warp shuffle instructions within a warp and using shared memory across warps. A single thread per warp executes the traceback operation. The size of the CIGAR string is not known ahead of time, hence it is stored as a linked list in global memory.

## 3 Results

### 3.1 Evaluation methodology

We demonstrate the benefits of Scrooge (along with each of our three algorithmic improvements) using both CPU and GPU implementations by comparing it to the recent WFA lm (Eizenga and Paten 2022), WFA (Marco-Sola et al. 2020), KSW2 (Suzuki and Kasahara 2018) (the state-of-the-art aligner used in minimap2; Li 2018), Edlib (Šošić and Šikić 2017) [the state-of-the-art implementation of Myers’ bitvector algorithm (Myers 1999) used in Medaka (https://github.com/nanoporetech/medaka) and Dysgu (Cleal and Baird 2022)], CUDASW++3.0 (Liu et al. 2013), Darwin-GPU (Ahmed et al. 2020), and our CPU and GPU implementations of the GenASM algorithm.

We evaluate the throughput and accuracy of Scrooge via three classes of experiments. First, we compare the throughputs of all evaluated tools and show that Scrooge outperforms state-of-the-art aligners. Second, we evaluate the throughput benefits of Scrooge’s algorithmic improvements and its sensitivity to different choices for W and O. Third, we evaluate Scrooge’s accuracy. We define throughput as the number of pairwise sequence alignments per second for a given dataset.

We run all CPU evaluations on a dual-socket Intel Xeon Gold 5118 (2× 12 physical cores, 2× 24 logical cores) (Intel 2017) at 3.2 GHz with 196GiB DDR4 RAM. We run all GPU evaluations on an NVIDIA A6000 (NVIDIA 2020). We repeat all CPU and GPU experiments 10 times and 5 times, respectively, and average the results.

#### 3.1.1 Datasets

We simulate 115 240 PacBio reads from the human genome using PBSIM2 (Ono et al. 2020), each of length 10 kilobases and with a target error rate of 5%. We obtain the ground truth location in the reference genome, and the alignment (CIGAR string) of each read from PBSIM2, thus obtaining 115 240 candidate pairs for our *long read groundtruth* dataset. We map 500 of the simulated PacBio reads to the human genome using minimap2 (Li 2018) and obtain all chains (candidate locations) it generates using the -P flag, 138 929 locations in total. This constitutes our *long read* dataset. We map 100 000 Illumina short reads from the dataset with accession number SRR13278681 to the human genome using minimap2 (Li 2018) and obtain all chains (candidate locations) it generates using the -P flag, 9 612 222 locations in total. This constitutes our *short read* dataset. We show further statistics of the datasets in Section 2 of the Supplementary Materials, including error, error rate, and sequence length distributions. The exact datasets and command lines that produced all our results, including those in the Supplementary Materials, are available at our GitHub repository: https://github.com/cmu-safari/Scrooge.

### 3.2 Throughput

**Figure 6.**
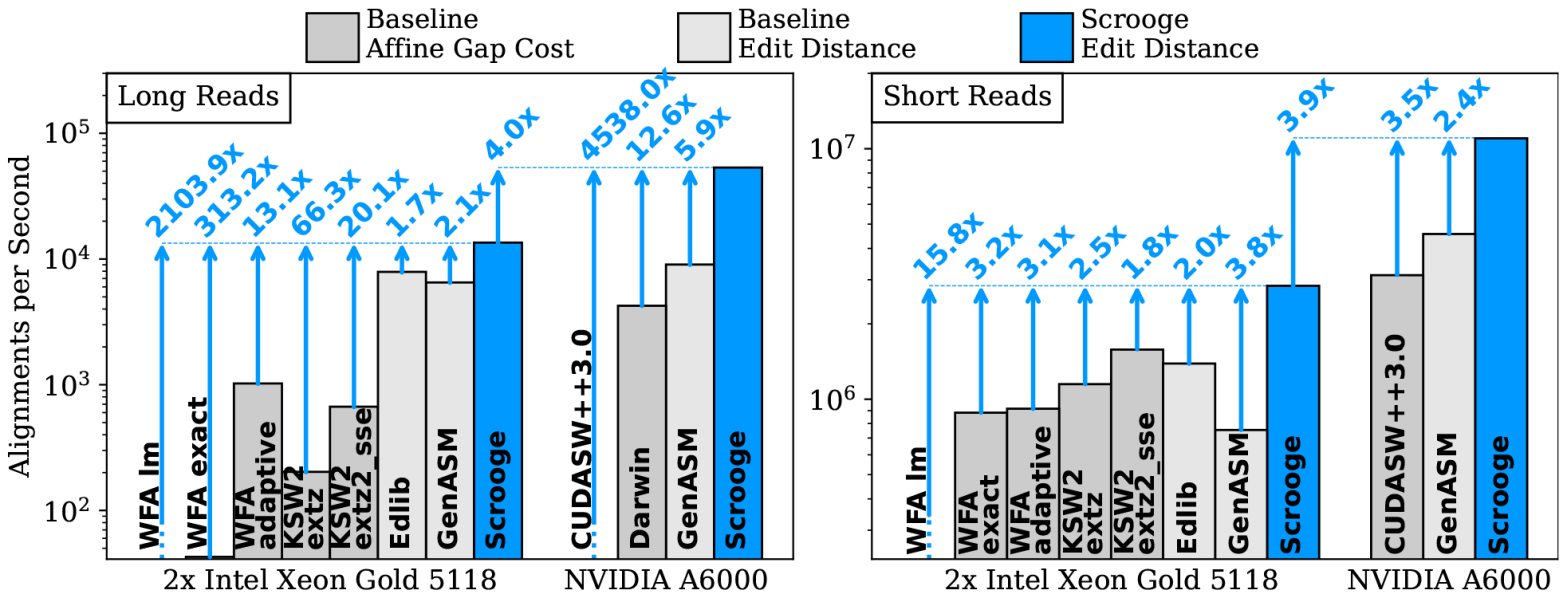
Scrooge’s alignment throughput relative to various CPU and GPU baselines.

We run the CPU-based tools using 48 threads. We set the band width (i.e. the edit distance threshold) of Edlib and KSW2 to 15% of the read length. We configure WFA-adaptive as recommended by its authors. We take the fastest configuration from a parameter sweep for Darwin-GPU. For a meaningful comparison, we ensure that Darwin-GPUs alignment component fully aligns all sequence pairs. We explain our changes to Darwin-GPU in Section 7 of the Supplementary Materials. We empirically configure Scrooge’s CPU and GPU implementations with W = 64, O = 33 for the long read dataset and W = 32, O = 17 for the short read dataset, and enable the combinations of improvements that yield the best throughput. The exact function calls and parameters we used for each tool can be found in our GitHub repository and in Section 7 of the Supplementary Materials. Figure 6 shows that Scrooge significantly speeds up the alignment of long and short reads over *all* baselines. In particular, the CPU implementation of Scrooge has 2.1× higher throughput (i.e. pairwise sequence alignments per second) than our CPU implementation of GenASM for long reads and 3.8× higher throughput for short reads. The GPU implementation of Scrooge has 5.9× higher throughput than our GPU implementation of GenASM for long reads and 2.4× higher throughput for short reads. The CPU and GPU speedups over GenASM are entirely due to Scrooge’s algorithmic improvements (i.e. SENE, DENT, ET) since our Scrooge and GenASM implementations are similarly optimized.

Note that WFA, KSW2, CUDASW++3.0, and Darwin solve a more general formulation of the alignment problem with affine gap scores (Gotoh 1982). This puts them at a performance disadvantage. In contrast, Edlib (Šošić and Šikić 2017), GenASM (Senol Cali et al. 2020), and Scrooge solve a less general but more efficient formulation of the alignment problem with unit costs (edit distance or Levenshtein distance; Levenshtein 1966). We list the capabilities of each tool in Section 6 of the Supplementary Materials.

### 3.3 Thread scaling

**Figure 7.**
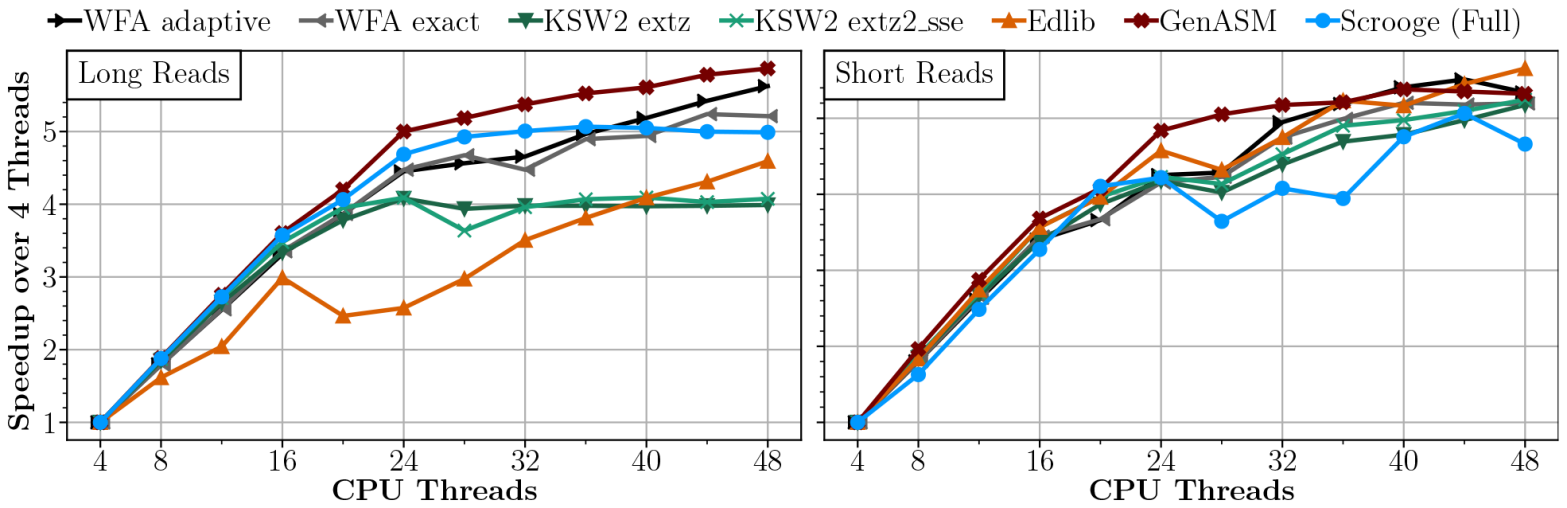
Speedup of each CPU tool as the number of CPU threads increases.

We explore the scaling of each CPU tool as the number of CPU threads increases. For each evaluated CPU tool, we sweep the number of CPU threads and measure the throughput on the long read and short read datasets. Figure 7 shows the results normalized to each tool’s throughput with four threads (for readability). We make three key observations. First, most tools scale almost linearly up to 24 threads for both datasets, but do not scale significantly from 24 to 48 threads. The system we perform our experiments on has 24 physical cores and 48 logical cores (Intel 2017), thus we hypothesize that the tools do not benefit from simultaneous multithreading (*Hyper-Threading* in Intel terminology) (Marr et al. 2002) due to the low latencies of simple arithmetic and bitwise instructions (Fog 2021), which is what the underlying alignment algorithms of the tools primarily consist of. Second, we observe that Edlib’s performance *decreases* from 16 to 20 threads in the long read dataset. Since this does not occur in the short read dataset, we hypothesize that Edlib suffers from cache thrashing in the long read dataset and that the data fits into the cache for the short read dataset. Third, we observe that both evaluated functions of KSW2 do not scale at all past 24 threads in the long read dataset. We hypothesize that KSW2 is bandwidth-bound in this case.

### 3.4 Sensitivity analysis

We explore the throughput benefits of our algorithmic improvements in parameter sweeps over (i) the number of GPU and CPU threads, (ii) the window size (W) parameter, and (iii) the window overlap (O) parameter.

**Figure 8.**
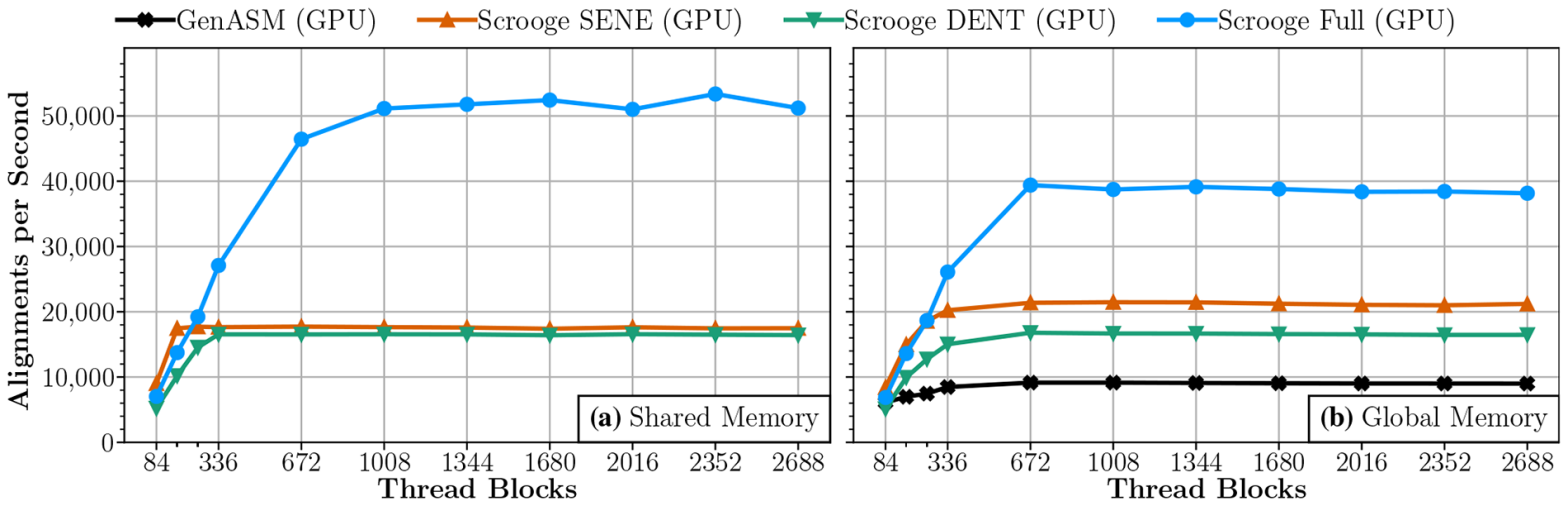
Scaling experiments of our GPU implementation with W = 64, O = 33, when the DP table placed in (a) shared memory and (b) global memory.

**GPU threads.** First, we run a scaling experiment on a GPU for GenASM, Scrooge with the SENE improvement, Scrooge with the DENT improvement, and Scrooge with all three proposed improvements, with the DP table placed in either shared memory (Fig. 8a) or global memory (Fig. 8b). Based on Fig. 8, we make five observations. First, we observe that SENE and DENT individually improve performance when the DP table is placed in either shared or global memory. Second, we observe that SENE, DENT, and ET can be combined for greater benefits. Third, we observe that placing the DP table in shared (on-chip) memory achieves the best performance, but only when both proposed memory footprint improvements (i.e. SENE and DENT) are applied. This is because only with SENE and DENT is the memory footprint small enough to keep sufficiently many problem instances in the shared memory to utilize the compute resources in each SM (see Section 2.3.2) well. Fourth, in configurations where the memory footprint is not reduced sufficiently (e.g. with only DENT or SENE), using global (off-chip) memory can be faster than using shared memory, because global memory has sufficient capacity to fit many problem instances, utilizing compute resources better than shared memory despite the global memory’s limited bandwidth. Finally, we observe that the baseline GenASM algorithm cannot run using shared memory at all, although we showed in Section 2.3.2 that a single instance of the baseline DP table has a footprint of 98.5KiB and thus should use fit into the 99KiB of shared memory. This is because our implementation requires some additional memory, such as for communication between processing elements. Thus, we cannot fit even a single instance into shared memory with GenASM.

We ran the experiment for all seven possible combinations of our three improvements (i.e. SENE, DENT, and ET). Full results are shown in Section 11 of the Supplementary Materials. In particular, we observe no significant benefits for GPUs from ET, which is why we omit it in Fig. 8 for readability.

**Figure 9.**
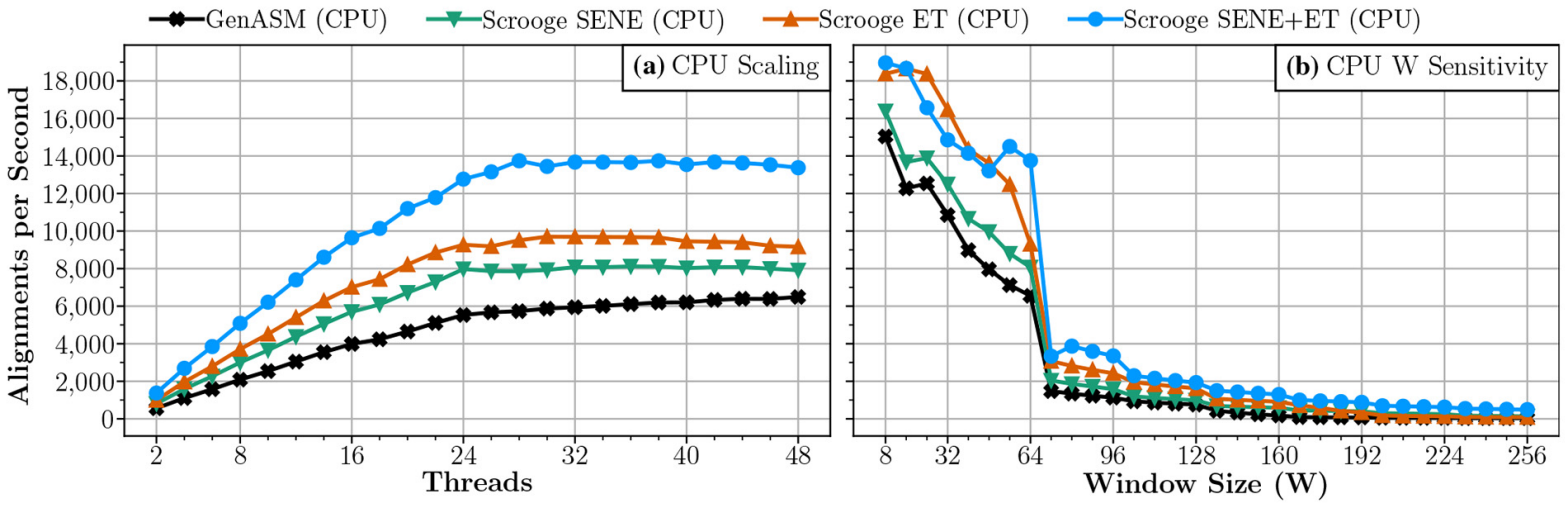
(a) Scaling and (b) sensitivity to window size of our CPU implementation.

**CPU threads.** We run a similar scaling experiment on a CPU for GenASM, Scrooge with the SENE improvement, Scrooge with the ET improvement, and Scrooge with the SENE and ET improvements. From Fig. 9a, we make three observations: First, we observe that ET improves performance significantly. This contrasts with our GPU implementation, where ET did not show significant benefits. This is because our CPU implementation builds the DP table R row-wise, while our GPU implementation builds R diagonal-wise (see Section 2.4.3). Second, SENE improves performance consistently, but less significantly than in the GPU case. This is because modern CPUs have relatively large on-chip cache capacities (e.g. 1MiB L2 cache per core on the Xeon Gold 5118 we evaluated on Intel 2017). Thus, the DP table easily fits into the L2 cache even without Scrooge’s algorithmic improvements, and hence reducing the memory footprint is not as important. Third, Scrooge scales linearly up to 24 threads but does not scale at all from 24 to 48 threads, a trend we observe for all evaluated tools (see Section 3.3).

We ran the experiment for all seven possible combinations of our three improvements (i.e. SENE, DENT, and ET). Full results are shown in Section 12 of the Supplementary Materials. In particular, we observe no significant benefits for CPUs from DENT, and in some cases even a slowdown, which is why we omit it in Fig. 9 for readability.

The three key takeaways from these experiments are that: (i) the SENE and DENT memory improvements yield significant benefits if performance is limited by memory bandwidth or capacity (e.g. in the GPU experiment), (ii) some of the algorithmic improvements can cause slight performance loss in practice (e.g. DENT in the CPU experiment), and (iii) the ideal combination of improvements depends on the computation platform (e.g. the available on-chip cache capacity) and the exact implementation (e.g. row-wise or diagonal-wise).

**Window size (W) and overlap (O).** We explore the sensitivity of Scrooge’s throughput to the window size parameter W (see Section 2.2.3) on CPUs. We vary W and set O = W//2 + 1. Note that larger W improves accuracy (see Section 2.2.3).

From the CPU results in Fig. 9b we make two observations: First, we observe that performance generally reduces as W increases. This is because the number of calculated bits per window increases cubically with increasing W. Second, we observe a sudden throughput dropoff when W increases past 64. This is because the word size of the Xeon Gold 5118 CPU is 64 bits; thus, if W> 64, each bitvector operation has to be emulated using multiple word-sized machine instructions. This emulation is conceptually simple (e.g. carry over shifted bits) but requires several additional instructions, causing the performance dropoff. For example, in our implementation, a single 65-bit left shift is performed using two 64-bit left shifts, a 64-bit right shift, and a bitwise or operation.

We repeat the same study on a GPU and observe the same trends: Increasing W reduces performance, and if the bitvectors are longer than the machine word (32 bits on the evaluated GPU), bit operations become significantly more expensive. We plot the GPU results and give detailed explanations in Section 4 of the Supplementary Materials.

We repeat a similar study for the window overlap (O) in Section 5 of the Supplementary Materials. We observe that as O increases, performance generally reduces. However, with Scrooge’s optimizations, larger values of O can sometimes *increase* performance. O = 33 gives the best result. Thus, we choose it as the default operating point of Scrooge for CPUs and GPUs.

### 3.5 Area and power consumption of an ASIC implementation

The GenASM ASIC designed in Senol Cali et al. (2020) uses a large on-chip scratchpad to store bitvectors for traceback. This scratchpad alone accounts for 0.256 mm^2^ (76%) of silicon area and 0.055 W (54%) of power out of a total of 0.334 mm^2^ and 0.101 *W* per accelerator core. Our proposed algorithmic improvements can be applied to that ASIC design through minor modifications. We estimate the area and power cost of such an ASIC implementation of Scrooge analytically as follows:

1. We start with the DC-logic, DC-SRAM, and TB-logic area and power numbers reported in Senol Cali et al. (2020)
2. We estimate Scrooge’s TB-SRAM area and power cost with CACTI 7 (Balasubramonian et al. 2017), as in Senol Cali et al. (2020), but with Scrooge’s reduced memory footprint and data movement numbers.
3. We account for the logic overhead of SENE by adding the area and power of a single DC processing element (Senol Cali et al. 2020) to the traceback (TB) logic cost, which accounts for recomputing edges during traceback. We assume no overhead for SENE during the construction of R, since the ANDed bitvectors are already computed.
4. We assume no overheads for applying DENT since it simply masks out bits when storing the bitvectors, which is trivial in hardware.

Table 1 lists the area and power breakdowns obtained with this methodology, and the breakdown of (Senol Cali et al. 2020) as a comparison point. In particular, we observe a 3.6× reduction in chip area and a 2.1× reduction in chip power consumption, while maintaining the same throughput. These improvements come from (i) the reduced TB SRAM capacity, and (ii) the reduced TB SRAM bandwidth.

**Table 1.** Estimated area and power of a Scrooge ASIC with W = 64 and O = 33.

	Area (${\text{mm}}^{2}$)					Power (*W*)				
ASIC implementation	DC logic	TB logic	DC SRAM	TB SRAM	Total	DC logic	TB logic	DC SRAM	TB SRAM	Total
Senol Cali *et al.* (2020)	0.049	0.016	0.013	0.256	0.334	0.033	0.004	0.009	0.055	0.101
Scrooge	0.049	0.016	0.013	0.014	0.093	0.033	0.004	0.009	0.003	0.049

The key takeaway from this estimate is that Scrooge’s algorithmic improvements (i) are directly applicable to and (ii) yield significant benefits over an ASIC implementation of GenASM.

### 3.6 Accuracy

The GenASM algorithm (Senol Cali et al. 2020), which Scrooge is based on, is a greedy heuristic algorithm, as explained in Section 2.2. Our improvements do *not* introduce additional inaccuracy. In fact, Scrooge’s default operating point of W = 64 O = 33 *increases* accuracy (see Section 2.2.3) over GenASM’s default operating point of W = 64 O = 24 (Senol Cali et al. 2020). The following analysis explores the accuracy of both Scrooge and GenASM across different operating points. At any given operating point, Scrooge produces the same alignments (and hence accuracy) as GenASM at that operating point. We run three types of experiments. First, we evaluate the alignment quality of Scrooge compared to all evaluated baseline tools. Second, we explore in detail the sensitivity of accuracy to the window size W. Third, we explore in detail the sensitivity of accuracy to the window overlap O.

**Figure 10.**
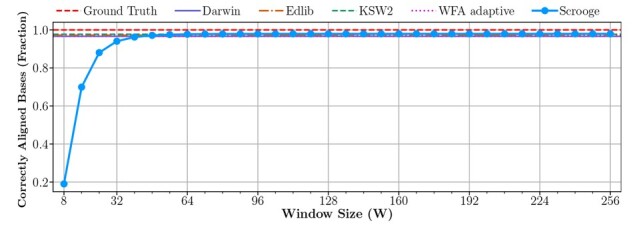
Fraction of correctly aligned bases according to the ground truth alignments in the long read groundtruth dataset.

**Alignment quality compared to baseline tools.** We explore the quality of the alignments (CIGAR strings) generated by Scrooge, compared to the baseline tools. To measure alignment quality, we count the number of correctly aligned bases according to the ground truth alignments reported by the PBSIM2 simulator for the long read groundtruth dataset. For Scrooge we repeat the evaluation for multiple values of W and set O = W//2 + 1. We make three observations from Fig. 10. First, the number of bases correctly aligned by Scrooge increases as the window size W increases. Second, Scrooge correctly aligns approximately the same number of bases as all of the baselines if W≥ 64. Third, no tool can consistently produce the exact ground truth alignment. By manually inspecting such misalignments of each tool, we determine this is because of two reasons. First, indels in homopolymers are ambiguous and cannot reliably be retrieved with any aligner. Second, sometimes the ground truth alignment is suboptimal in terms of alignment score and/or edit distance. In these cases, the aligners’ goal of finding the optimal scoring alignment produces high-scoring but wrong alignments.

We explore the sensitivity of Scrooge’s accuracy to the window size parameter W (see Section 2.2.3). We analyze the accuracy compared to optimal edit distance solutions, such as Edlib (Šošić and Šikić 2017). We evaluate the generated alignments based on minimap2’s default affine gap scoring model. We vary W and set O = W//2 + 1. For each experiment, we record the 0.5, 0.1, 0.01, and 0.001 percentile alignment scores (i.e. for a dataset of 1000 pairs, the 0.5 percentile would be the 500th worst alignment score, the 0.01 percentile would be the 10th worst alignment score) of Scrooge and GenASM (which produce the same results for the same choice of W and O) and compare to Edlib as an ideal upper bound.

**Figure 11.**
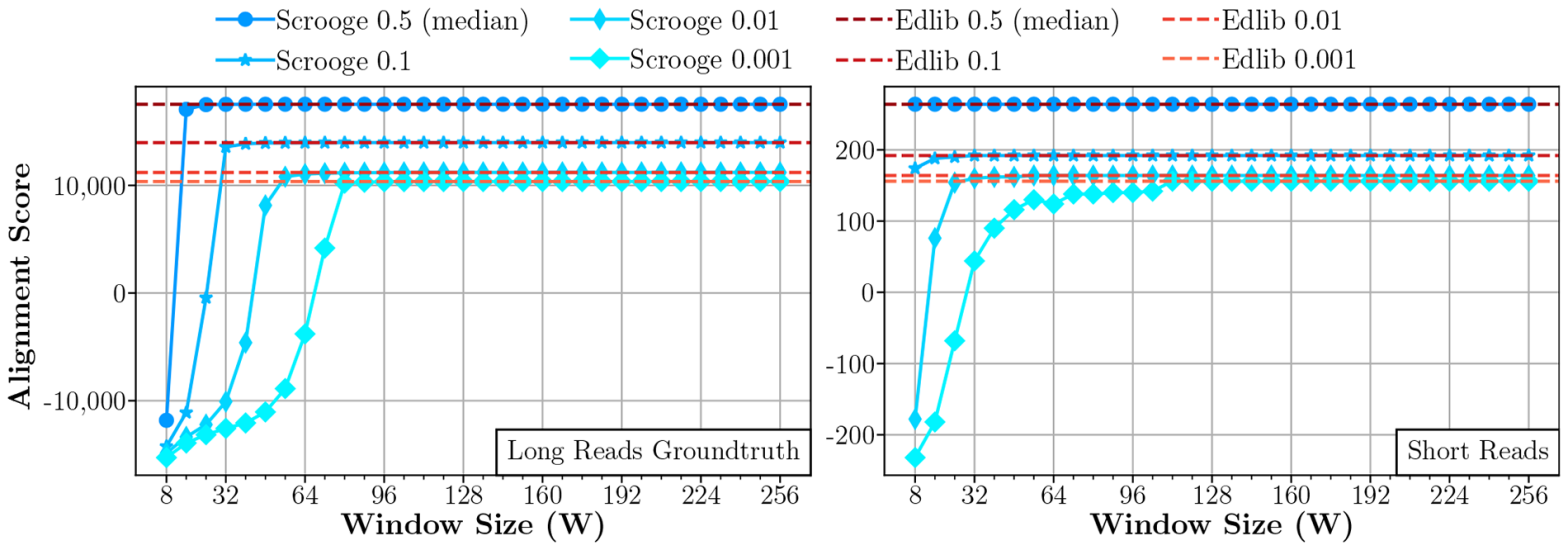
Sensitivity of Scrooge’s accuracy to W. We show the achieved alignment score of the 0.001, 0.01, 0.1, and 0.5 (median) quantiles, and compare to Edlib as an upper bound for the accuracy achievable with the edit distance metric.

**Sensitivity to window size (W).** From Fig. 11, we make three observations: First, accuracy depends on the dataset. Second, small window sizes are sufficient for Scrooge and GenASM to find the optimal edit distance alignment for *most* of the sequence pairs. For example, the median alignment score is already optimal at W = 32 for the long read groundtruth dataset and at W = 8 for the short read dataset. Third, to find the optimal alignment for a few worst-case pairs, large window sizes are required: For example, the optimal alignment for the 0.001 percentile in the long read groundtruth dataset is only found for W≥ 80. We manually inspect several of these ‘difficult’ sequence pairs to find the reason for their apparent difficulty. We observe sequence pairs are aligned poorly if they contain extremely noisy and repetitive sub-sequences. However, these pairs *will* be aligned optimally if the window size is larger than the length of the noisy sub-sequences. We illustrate this observation with an example sequence pair from the long read groundtruth dataset in Section 13 of the Supplementary Materials.

**Sensitivity to window overlap (O).** We explore the sensitivity of Scrooge’s accuracy to the window overlap parameter O (see Section 2.2.3). We sweep over O and run experiments for each W∈{32,64,96,128}. For each experiment, we record the 0.01 percentile alignment score of Scrooge/GenASM and compare to Edlib as an ideal upper bound.

**Figure 12.**
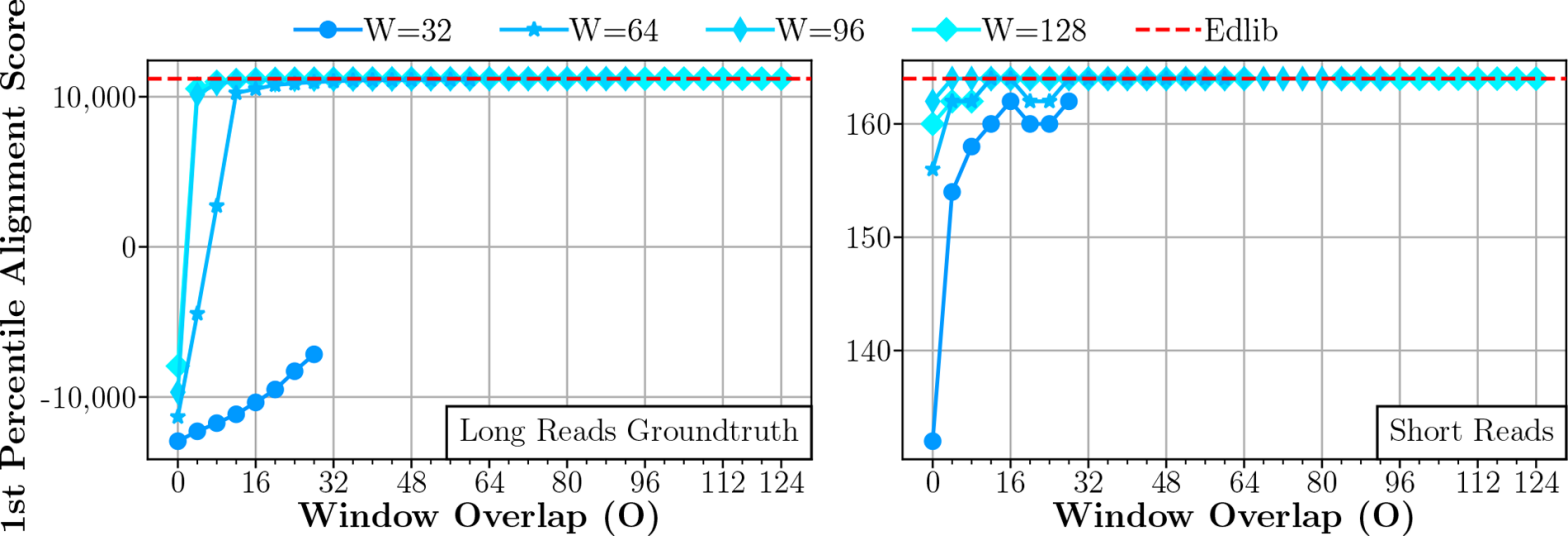
Sensitivity of accuracy to O, reporting the first percentile (worst 1%) alignment score for each configuration. Edlib is an upper bound for the scores achievable with the edit distance metric.

From Fig. 12, we make two observations: First, accuracy improves as O increases. Second, we observe that W and O need to be *balanced* to achieve good accuracy. For example, the accuracy loss of a too small W = 32 for the long read groundtruth dataset cannot be overcome with even large O = 30. Similarly, choosing O close to 0 hurts accuracy for both datasets, even when W is large.

The two key takeaways from these experiments are that (i) W and O need to be chosen per dataset, and (ii) W and O should be increased or reduced together for the best accuracy.

## 4 Discussion and conclusion

To our knowledge, this is the first paper to: (i) demonstrate the computational inefficiencies in the GenASM algorithm, (ii) address them with three improvements in our new Scrooge algorithm, (iii) rigorously demonstrate the computational benefits of Scrooge over GenASM for CPU, GPU, ASIC implementations, and (iv) rigorously analyze the accuracy of GenASM and Scrooge under multiple different configurations.

We have already extensively compared to WFA (Marco-Sola et al. 2020), KSW2 (Li 2018; Suzuki and Kasahara 2018), Edlib (Šošić and Šikić 2017), CUDASW++3.0 (Liu et al. 2013), and Darwin-GPU (Ahmed et al. 2020). Several other works accelerate sequence alignment: NVBIO (https://github.com/NVlabs/nvbio) is a multipurpose library for accelerating bioinformatics applications using GPUs, but is no longer maintained. Gasal2 (Ahmed et al. 2019) is a recent GPU aligner limited to short reads. CUDAlign4.0 (de Oliveira Sandes et al. 2016) can efficiently align a single pair of extremely long (chromosome-sized) sequences, with use cases such as whole genome alignment. Adept (Awan et al. 2020) is a recent GPU aligner for short and long reads but does not support traceback, i.e. only reports the alignment score.

The Darwin accelerator (Turakhia et al. 2018) implements a Smith–Waterman–Gotoh accelerator for long reads using a similar greedy strategy to GenASM called *tiling*. We have compared Scrooge to the GPU implementation of this algorithm, Darwin-GPU. GenASM, Scrooge, and Darwin demonstrate the significant benefits of greedy algorithms, based on which there are at least two interesting future directions to explore. First, a suitability study of different algorithms to greedy heuristics, such as Myers’ bitvector algorithm (Myers 1999), Hyyrö’s banded bitvector algorithm (Hyyrö 2003), or the recently proposed wavefront algorithm (Marco-Sola et al. 2020). Second, an exploration of the effectiveness of our algorithmic improvements for other implementations of greedy windowing or tiling, like Darwin. We believe the DENT improvement can be applied directly to Darwin.

We have demonstrated the computational benefits of Scrooge over a variety of state-of-the-art baselines for both commodity hardware (i.e. CPUs and GPUs) and custom hardware (i.e. ASICs). We have demonstrated the accuracy of Scrooge for multiple datasets. We conclude that Scrooge has clear benefits across a wide range of computing platforms.

## Supplementary Material

btad151_Supplementary_Data
