# Supplementary material for: Scrooge: a fast and memory-frugal genomic sequence aligner for CPUs, GPUs, and ASICs
Source: Bioinformatics. 2023 Mar 24;39(5):btad151. doi: 10.1093/bioinformatics/btad151 (PMC10191611; doi:10.1093/bioinformatics/btad151)
Supplement: btad151_Supplementary_Data [file btad151_supplementary_data.pdf]

Supplementary Materials

1. DENT extended to rows

Since the W-O characters that are traced back can incur an edit cost of at most W-O, traceback also only reads at most W-O+1 rows starting from row distance(pattern, text). Thus the first max(0, distance(pattern, text) - (W-O+1)) do not need to be stored. Supplementary Figure 1 visualizes the concept of DENT extended to rows.

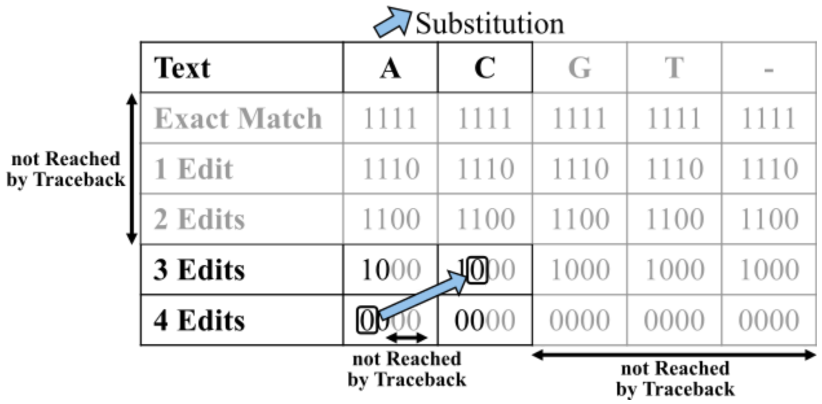

Suppl. Fig. 1: When extended to rows, DENT can also discard rows with low edit distances that can never be reached.

Note that determining the correct rows to store is dependent on distance(pattern, text), which is unknown ahead of the construction on R. If we build R row-wise we can always store the most recent W-O+1 rows of R and overwrite the oldest when we run out of space. To ensure we do not overwrite useful entries, we can apply the Early Termination improvement (Section 2.4.3) to stop as soon as we find distance(pattern, text).

Extended DENT (with SENE) provisions space for W-O+1 bits per entry, W-O+1 columns, and up to W-O+1 rows. For W=64 and O=33, this is 32^3 bits. SENE without dent would provision 65^2*64 bits, i.e., extended DENT improves the memory footprint by over 8x.

Extended DENT relies on row-wise computation and Early~Termination to not overwrite row entries required for traceback. This is not possible with the diagonal-wise computation, thus we only implement the weaker version of DENT, see Section 2.4.2. However, cases where extended DENT is useful can be easily imagined: Extended DENT needs row-wise computation, thus the program should be written in a sequential manner, as opposed to intra-task cooperative (which requires diagonal-wise computation). If that architecture then is constrained for on-chip memory capacity, extended DENT can address the issue.

2. Dataset Details

Supplementary Figures 2-7 show the edit and sequence length distributions for each dataset we used. The histograms were generated as follows:

1. Extract the sequence region pairs specified in the maf or paf file
2. Consider the length of the longer sequence in each pair as the pairs length
3. Align each pair with Edlib to obtain the edit distance, and divide it by the length of the pair
4. Draw the histograms

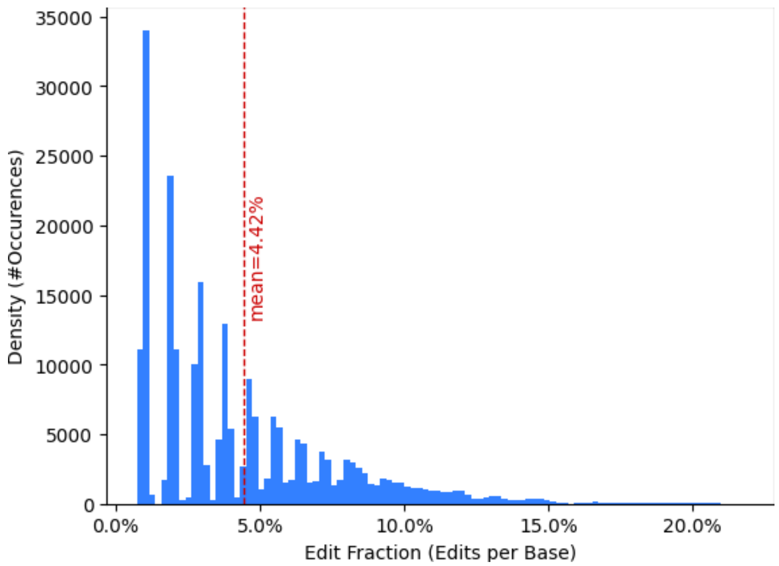

Suppl. Fig. 2: Histogram of edit distributions in the long read groundtruth dataset.

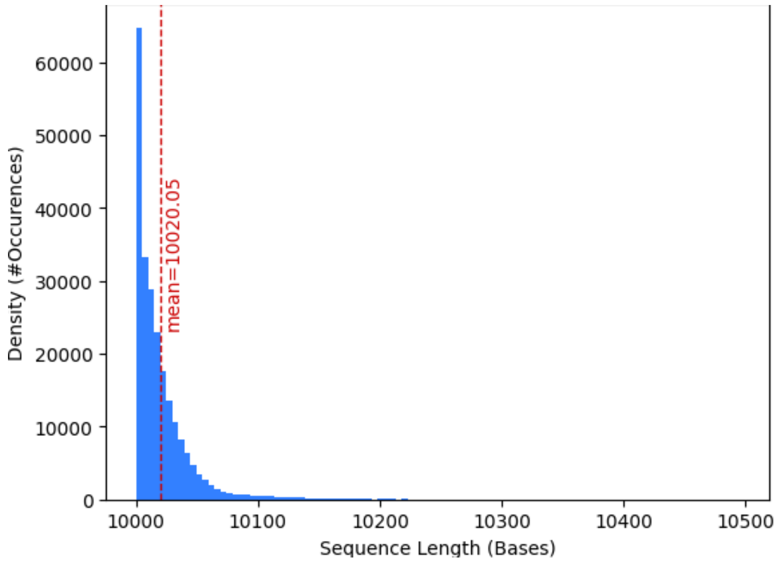

Suppl. Fig. 3: Histogram of the sequence lengths in thelong read groundtruth dataset.

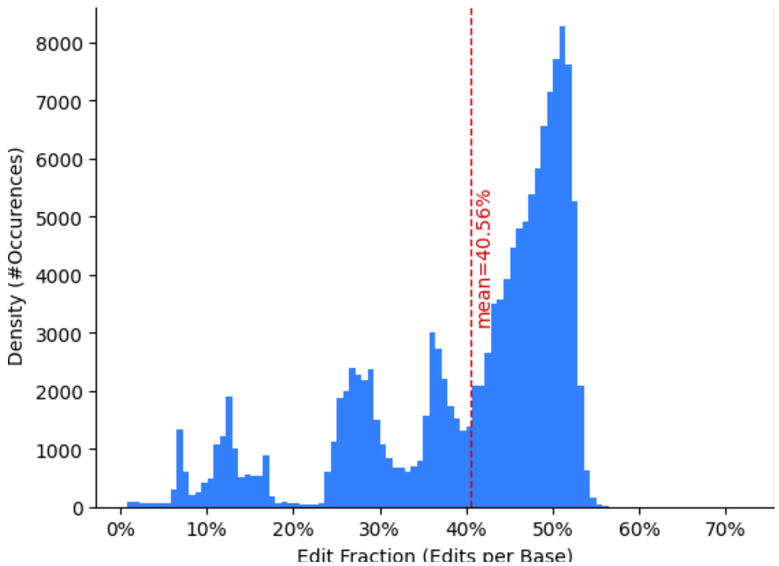

Suppl. Fig. 4: Histogram of edit distributions in the long read dataset.

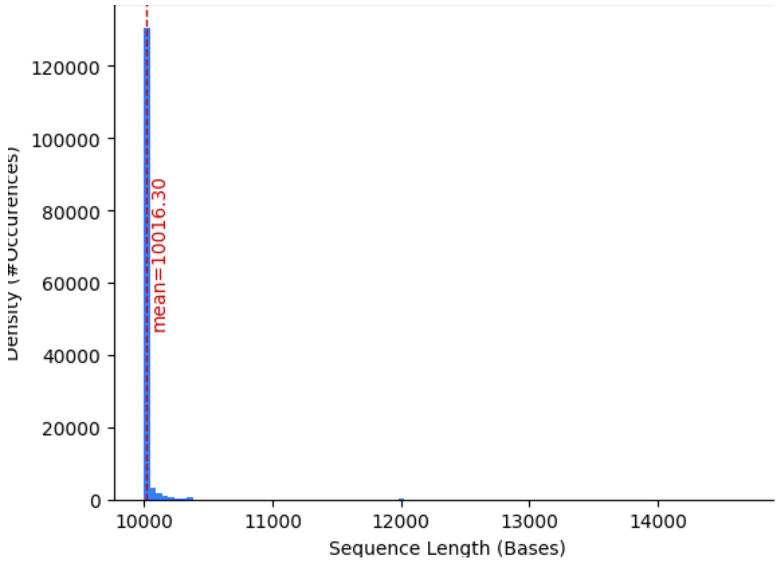

Suppl. Fig. 5: Histogram of the sequence lengths in the long read dataset.

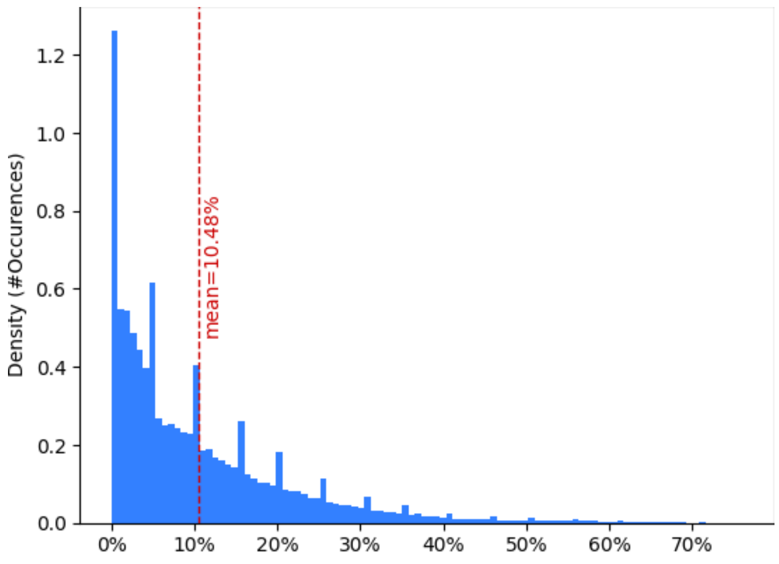

Suppl. Fig. 6: Histogram of edit distributions in the short read dataset.

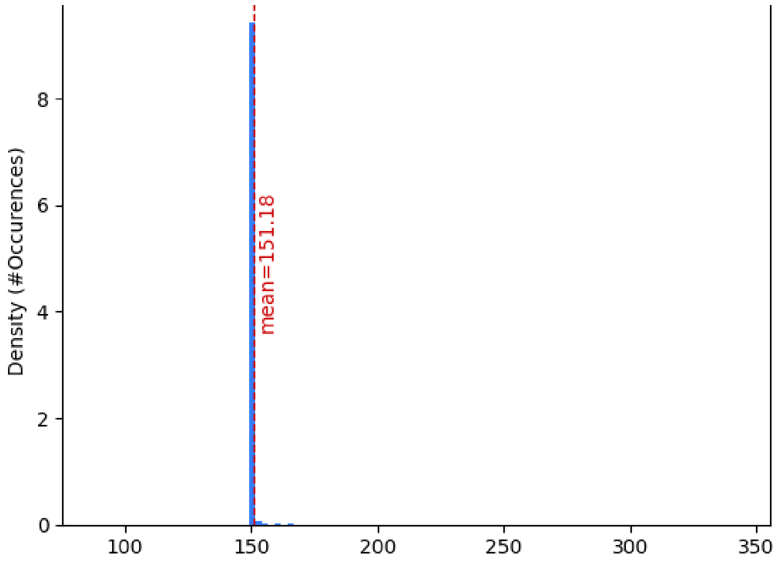

Suppl. Fig. 7: Histogram of the sequence lengths in the short read dataset.

3. Throughput Distributions

To understand the quality of our throughput results, we plot the throughput distributions of each evaluated tool as box plots. Supplementary Figure 8 shows the results. We observe that all throughput values are highly consistent.

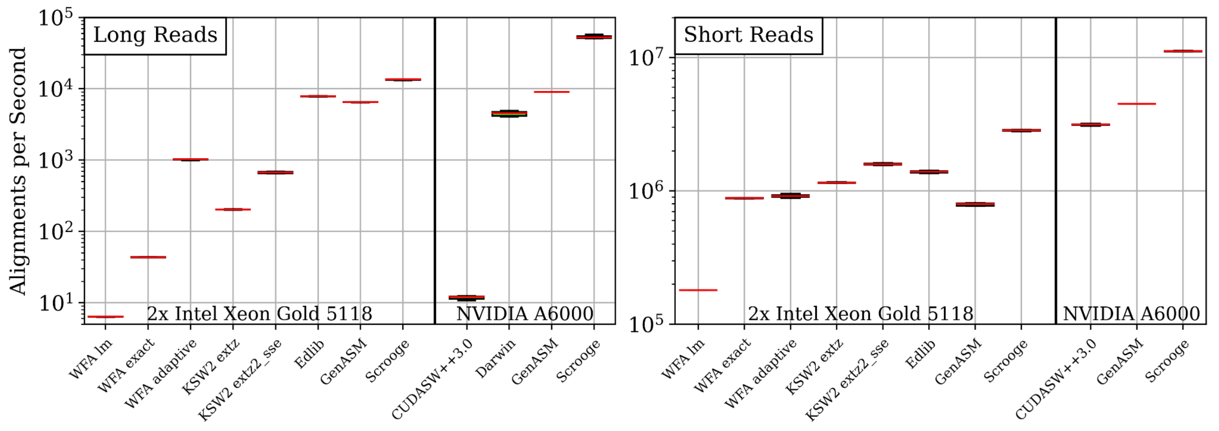

Suppl. Fig. 9: Throughput distributions of each evaluated tool. Each boxplot consists of the median as a red line, and the first and third quartiles as the lower and upper end of the box, respectively. The whiskers are 1.5 inter-quartile ranges down and up from the first and third quartile, respectively.

4. Throughput Sensitivity to Window Size (W) on GPU

We explore the sensitivity of Scrooge's throughput to the window size parameter W (Section 2.2.3) on GPUs. We vary W and set O=W//2+1. Note that larger W improve accuracy (Section 2.2.3). From the GPU results in Supplementary Figure 8 we make five observations: First, as on the CPU, performance generally reduces as W increases. Second, as on the CPU, there are sudden performance dropoffs whenever the window (and thus bitvector) size surpasses a multiple of the machine word size (32 bits on the tested GPU (NVIDIA, 2023)). Third, some configurations can run only for small W when the DP table R is placed in shared memory. This is because as W increases, the memory footprint of R increases cubically, and some configurations run out of shared memory. With global memory, the DP tables can occupy as much of the GPUs off-chip memory as needed, thus Scrooge can run even very large W, although the performance eventually becomes impractically low. Fourth, for very small W, shared memory can offer significantly better performance than global memory. This is because for smaller W the memory footprint per DP table is smaller, and more instances can be kept in shared memory, thus easily achieving high occupancy. In contrast, global memory remains bound by memory bandwidth, because the operational intensity is unchanged. Fifth, for small window sizes and shared memory it is beneficial to run without the memory improvements SENE and DENT. This corroborates the observation made for the CPU implementation: If memory capacity and bandwidth are not limiting factors, then the computational overheads of SENE and DENT overshadow their benefits. In contrast, when W is large, the memory improvements become increasingly important because of the inverse argument. The three key takeaways from these experiments are that (1) the ideal combination of improvements depends on the window size (W) parameter, (2) the memory improvements SENE and DENT memory improvements are increasingly beneficial as the window size increases, and (3) the window size W should be exactly or a small multiple of the machine word size for optimal throughput.

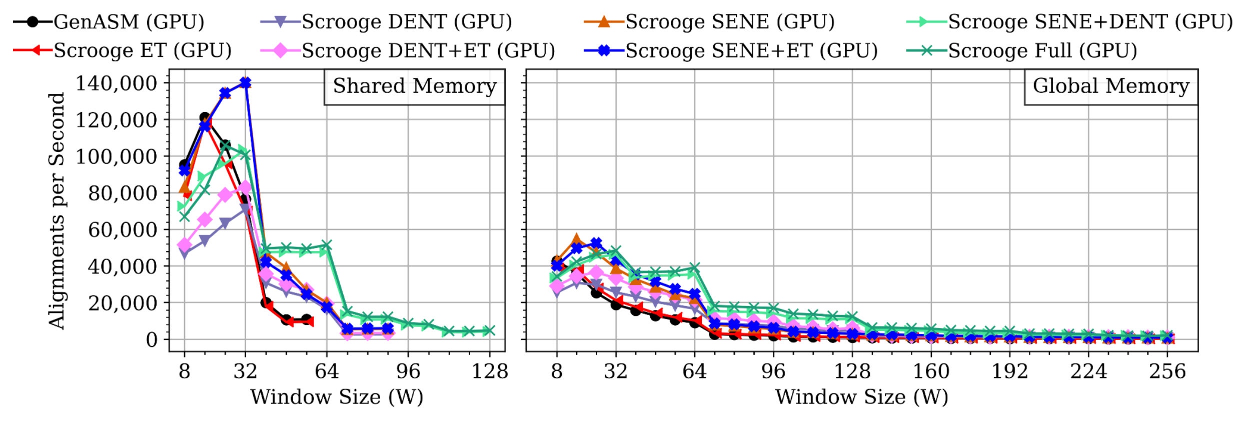

Suppl. Fig. 9: Performance of our GPU version with varying W and O=W//2+1.

5. Throughput Sensitivity to Window Overlap (O) on GPU

We explore the sensitivity of Scrooge's throughput to the window overlap parameter O (Section 2.2.3) on GPUs. We vary O and set W=64. Note that larger O improve accuracy (Section 2.2.3). From the GPU results in Supplementary Figure 9 we make two observations: First, when DENT is disabled, performance decreases strictly and smoothly as O increases. This is because the algorithm makes W-O characters progress per window, i.e. progress per window decreases linearly with increasing O. Second, we observe that with DENT enabled, performance can sometimes

increase as O increases, up to some limit. This is because DENT reduces the memory footprint of the DP table more as O increases. We observe a large and sudden increase in performance at O=34. This is because with DENT, each bitvector stored for traceback has size W-O+1, thus for O≥33 each stored bitvector fits into a single 32-bit machine word (NVIDIA, 2020). The key takeaway from this experiment is that a larger window overlap (O) can improve performance up to some limit. This is convenient because larger values of O also improve accuracy. In particular, we found for W=64 the optimal performance is attained by O=33, increasing both throughput and accuracy over the default operating point chosen by Senol Cali et al. (2020) of W=64 and O=24 for their accelerator.

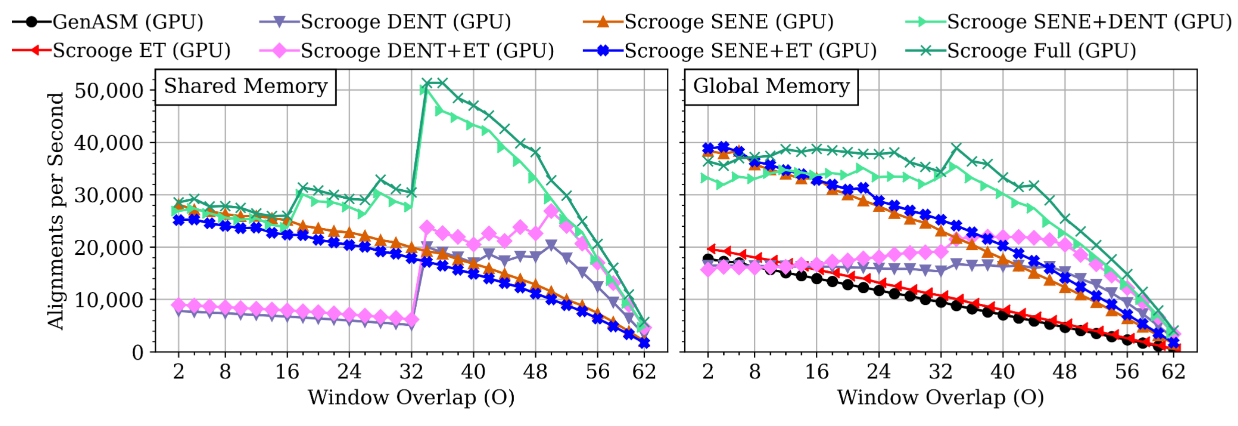

Suppl. Fig. 10: Performance of our GPU implementation with W=64 and varying O.

6. Tool Capabilities

Supplementary Table 1 lists the capabilities (scoring scheme, optimality guarantee, acceleration) of each evaluated tool variant.

Suppl. Tab. 1: Properties of each evaluated tool variant.				
**Tool**	**Supported Scoring Scheme**	**Banded?**	**Optimality Guarantee?**	**Acceleration**
WFA lm	One-piece Affine Gap	No	Yes	
WFA exact	One-piece Affine Gap	No	Yes	SSE2
WFA adaptive	One-piece Affine Gap	No	No	SSE2
KSW2 extz	One-piece Affine Gap	Yes	Yes	
KSW2 extz2_sse	One-piece Affine Gap	Yes	Yes	SSE2
Edlib	Edit Distance	Yes	Yes	
GenASM (CPU)	Edit Distance	No	No	
Scrooge (CPU)	Edit Distance	No	No	
CUADSW++3.0	One-piece Affine Gap	No	Yes	GPU
Darwin (GPU)	One-piece Affine Gap	No	No	GPU
GenASM (GPU)	Edit Distance	No	No	GPU
Scrooge (GPU)	Edit Distance	No	No	GPU

7. Tool Function Calls and Parameters

Supplementary Table 2 lists the exact function calls and parameters we used to evaluate each tool.

<table>
<tr><td colspan="3">Suppl. Tab. 2: Function calls and parameters for each evaluated tool variant.</td></tr>
<tr><th>Tool</th><th>Clear Text Parameters</th><th>Function Call</th></tr>
<tr><td>WFA lm</td><td>Scores=1,1,1,1</td><td><pre><code>wfalm::SWGScores scores;
scores.match = 1;
scores.gap_extend = 1;
scores.gap_open = 1;
scores.mismatch = 1;
wfalm::wavefront_align_low_mem(text, pattern, scores);</code></pre></td></tr>
<tr><td>WFA exact</td><td>Scores=0,1,1,1</td><td><pre><code>affine_penalties_t affine_penalties = {
    .match = 0,
    .mismatch = 1,
    .gap_opening = 1,
    .gap_extension = 1
};
mm_allocator_t* mm_allocator = mm_allocator_new(BUFFER_SIZE_8M);
affine_wavefronts_t* affine_wavefronts
    affine_wavefronts_new_complete(max_read_length,
    max_read_length*105/100, &affine_penalties, NULL, mm_allocator);
affine_wavefronts_align(affine_wavefronts, inputs[pair_idx].pattern,
    inputs[pair_idx].m, inputs[pair_idx].text, inputs[pair_idx].n);</code></pre></td></tr>
<tr><td>WFA adaptive</td><td>Scores=0,1,1,1
Min_Wavefront_Length=10
Max_Distance_Threshold=50</td><td>Runtime Evaluation:<pre><code>affine_penalties_t affine_penalties = {
    .match = 0,
    .mismatch = 1,
    .gap_opening = 1,
    .gap_extension = 1
};
const int min_wavefront_length = 10;
const int max_distance_threshold = 50;
mm_allocator_t* mm_allocator = mm_allocator_new(BUFFER_SIZE_8M);
affine_wavefronts_t* affine_wavefronts =
    affine_wavefronts_new_reduced(max_read_length,
    max_read_length*105/100, &affine_penalties, min_wavefront_length,
    max_distance_threshold, NULL, mm_allocator);
affine_wavefronts_align(affine_wavefronts, inputs[pair_idx].pattern,
    inputs[pair_idx].m, inputs[pair_idx].text, inputs[pair_idx].n);</code></pre></td></tr>
</table>

<table>
<tr><td></td><td>Scores=0,4,4,2
Min_Wavefront_Length=10
Max_Distance_Threshold=50</td><td>Accuracy Evaluation:

```
affine_penalties_t affine_penalties = {
    .match = 0,
    .mismatch = 4,
    .gap_opening = 4,
    .gap_extension = 2
};
const int min_wavefront_length = 10;
const int max_distance_threshold = 50;
mm_allocator_t* mm_allocator = mm_allocator_new(BUFFER_SIZE_8M);
affine_wavefronts_t* affine_wavefronts =
    affine_wavefronts_new_reduced(max_read_length,
    max_read_length*105/100, &affine_penalties, min_wavefront_length,
    max_distance_threshold, NULL, mm_allocator);
affine_wavefronts_align(affine_wavefronts, inputs[pair_idx].pattern,
    inputs[pair_idx].m, inputs[pair_idx].text, inputs[pair_idx].n);
```
</td></tr>
<tr><td>KSW2 extz</td><td>Scores=2,4,4,2
Band Width=15%</td><td>

```
int8_t a = 2;
int8_t b = -4;
int8_t score_matrix[25] = { a,b,b,b,0, b,a,b,b,0, b,b,a,b,0, b,b,b,a,0,
    0,0,0,0,0 };
int gap_open = 4;
int gap_extend = 2;
ksw_extz(NULL, inputs[pair_idx].m, inputs[pair_idx].pattern,
    inputs[pair_idx].n, inputs[pair_idx].text, 5, score_matrix,
    gap_open, gap_extend, (int)(0.15*inputs[pair_idx].m), -1,
    KSW_EZ_EXTZ_ONLY, &ez);
```
</td></tr>
<tr><td>KSW2 extz2_sse</td><td>Scores=2,4,4,2
Band Width=15%</td><td>

```
int8_t a = 2;
int8_t b = -4;
int8_t score_matrix[25] = { a,b,b,b,0, b,a,b,b,0, b,b,a,b,0, b,b,b,a,0,
    0,0,0,0,0 };
int gap_open = 4;
int gap_extend = 2;
int end_bonus = 0;
ksw_extz2_sse(NULL, inputs[pair_idx].m, inputs[pair_idx].pattern,
    inputs[pair_idx].n, inputs[pair_idx].text, 5, score_matrix,
    gap_open, gap_extend, (int)(0.15*inputs[pair_idx].m), -1,
    end_bonus, KSW_EZ_EXTZ_ONLY, &ez);
```
</td></tr>
<tr><td>Edlib</td><td>Band Width=15%</td><td>

```
EdlibAlignConfig conf;
conf.k = (int)(0.15*inputs[pair_idx].m);
conf.mode = EDLIB_MODE_SHW;
conf.task = EDLIB_TASK_PATH;
conf.additionalEqualities = NULL;
conf.additionalEqualitiesLength = 0;
EdlibAlignResult res = edlibAlign(inputs[pair_idx].pattern,
    inputs[pair_idx].m, inputs[pair_idx].text, inputs[pair_idx].n,
    conf);
```
</td></tr>
</table>

GenASM (CPU)	W=64 O=33	`#define W 64`<br>`#define O 33`<br>`genasm_cpu::align_all(reference, reads, threads, &core_algorithm_ns);`
Scrooge (CPU)	W=64 O=33<br>SENE=True<br>DENT=False<br>EarlyTerm.=True	`#define W 64`<br>`#define O 33`<br>`#define STORE_ENTRIES_NOT_EDGES`<br>`#define EARLY_TERMINATION`<br>`genasm_cpu::align_all(reference, reads, threads, &core_algorithm_ns);`
CUADSW++3.0	Defaults	Standalone
Darwin (GPU)	T=320 O=120<br>(Default)	Standalone<br>By default, Darwin-GPU does not fully evaluate or align sequence pairs whose score drops to 0. While sensible in the context of a full mapper, for a fair comparison of pairwise sequence alignment throughput, we modified Darwin-GPU's pairwise sequence alignment function to fully evaluate each sequence pair. The relevant change can be found at https://github.com/CMU-SAFARI/Scrooge/blob/main/baseline_algorithms/darwin-gpu/cuda_header.h#L177
GenASM (GPU)	W=64 O=33	`#define W 64`<br>`#define O 33`<br>`genasm_cpu::align_all(reference, reads, &core_algorithm_ns);`
Scrooge (GPU)	W=64 O=33<br>SENE=True<br>DENT=True<br>EarlyTerm.=False	`#define W 64`<br>`#define O 33`<br>`#define STORE_ENTRIES_NOT_EDGES`<br>`#define DISCARD_ENTRIES_NOT_USED_BY_TRACEBACK`<br>`genasm_cpu::align_all(reference, reads, &core_algorithm_ns);`

8. Roofline Model Calculations

We analyze a single GenASM window of size W=64 (see Section 2.2.3). Since GenASM simply repeats the calculation of a window in a loop, this analysis is representative of the entire program.

8.1 GenASM Data Movement

Per window, GenASM generates W=64 intermediate bitvectors to initialize the topmost row of R (Line 11 of Algorithm 1). It also generates 4×64×64 intermediate bitvectors for the inner entries of R (Lines 13-17 of Algorithm 1), out of which 3×64×64 are stored for traceback (Section 2.2.2). Bitvectors have size W=64 bits each. We assume that the processing elements communicate neighbor entry values without memory accesses. This is a realistic assumption, for example, both the hardware accelerator in (Senol Cali et al., 2020), as well as our GPU implementation are implemented this way. Thus, each of these bitvectors is written to memory exactly once per window (during the construction of R) and not read again. We ignore the memory accesses for reading the input sequences and for traceback, as the consumed bandwidth is negligible relative to DP table construction. This yields data_movement = (64 + 3×64×64) × 64b = 98,816B = 96.5KiB.

8.2 GenASM Operational Intensity

The operational intensity is defined as the ratio of work done over data movement (Ofenbeck et al., 2014). We define work as the number of arithmetic and logic 64-bit integer operations (op), and data_movement as the number of bytes moved between registers and the memory hierarchy and scratchpad memory.

In each window, first, GenASM computes 65 entries to initialize the rightmost column, using 1 op each (Line 5). Second, it computes 64 entries to initialize the topmost row, using 2 op each (Line 11). Third, it computes 64×64 entries using 7 op each (Lines 13-17 of Algorithm 1). Thus, work = 65op + 64×2op + 64×64×7op = 28,865op. The GenASM algorithm's operational intensity is then I = work / data_movement ≈ 0.29 op/B .

8.3 Bandwidth and Compute Rooflines

NIVIDIA A6000 GPU. We abbreviate “streaming multiprocessor” as “SM”.

We obtain the A6000's *global* (off-chip) memory bandwidth from its datasheet, 768 GB/s.

We derive the *shared* (on-chip) memory bandwidth from the CUDA Programming Guide (NVIDIA, 2023) by multiplying the per-cycle-per-SM throughput (128B/cycle/SM) with the clock frequency (1.8GHz) and number of SMs (84), 19353.6GB/s.

We derive compute throughput from the CUDA Programming Guide (NVIDIA, 2023) by multiplying the per-cycle-per-SM throughput (32op/cycle/SM) with the clock frequency (1.8GHz) and number of SMs (84), 4838.4 Gop/s.

Intel Xeon 5118 CPU. We obtain the off-chip (DRAM) bandwidth by assuming 2,400MT/s DDR4 memory, multiplied by the bus width (8B) and maximum number of memory channels supported by the CPU (6), 115.2GB/s.

We obtain the cache bandwidths from the Intel Optimization Manual (Intel, 2023) by multiplying the respective per-cycle-per-core throughputs (L3: 15B/cycle/core, L2: 52B/cycle/core, L1: 133B/cycle/core) with frequency (2.3GHz) and number of cores (12), L3: 414GB/s, L2: 1,435.2GB/s, L1: 3,670.8GB/s.

We obtain the compute throughputs from the Intel Optimization Manual (Intel, 2023) by multiplying the respective per-cycle-per-core throughputs (Scalar: 4op/cycle/core, AVX512: 16op/cycle/core) with frequency (2.3GHz) and number of cores (12), Scalar: 110.4Gop/s, AVX512: 441.6Gop/s.

9. GenASM Memory Footprint Calculation

GenASM stores W=64 intermediate bitvectors to initialize the topmost row of R (Line 11 of Algorithm 1). It also generates 4×64×64 intermediate bitvectors for the inner entries of R (Lines 13-17 of Algorithm 1), out of which 3×64×64 are stored for traceback (Section 2.2.2). Bitvectors have size W=64 bits each. This yields working_set_memory_footprint = (64 + 3×64×64) × 64b = 98,816B = 96.5KiB.

10. Expected Edit Distance for Random String Pairs

Lemma S1. The expected edit distance between an uncorrelated random string pair of length W over an alphabet of size |Σ| is at most W*(|Σ|-1)/|Σ|.
Proof. We prove Lemma S1 in two steps. First, we calculate the expected Hamming distance between an uncorrelated random string pair. Second, we prove that the edit distance of a string pair of equal length is at most its Hamming distance.

From Lemma S1 follows in particular that for a 4-character DNA alphabet, the expected edit distance between an uncorrelated random string pair of length W is at most W*(4-1)/4=3*W/4.

Lemma S2. The expected Hamming distance between an uncorrelated random string pair of length W over an alphabet of size |Σ| is W*(|Σ|-1)/|Σ|.
Proof. The Hamming distance between two strings is defined as the number of locations where they differ (Hamming, 1950). For two uncorrelated random strings over an alphabet of size |Σ|, the probability that they differ at a given location is P_{differ}=(|Σ|-1)/|Σ|, and the expected number of differences at a single location is E[differ]=P_{differ}=(|Σ|-1)/|Σ|. For W possible locations in two strings of length W is then E[differeces]=W*E[differ]=W*(|Σ|-1)/|Σ|=E[Hamming].

Lemma S3. The Hamming distance of a pair of strings of equal length is an upper bound on its edit distance.
Proof. The edit distance of a string pair is defined as the smallest number of single-character insertions, deletions, and substitutions to convert one into the other (Levenshtein, 1966). Suppose the Hamming distance of an equal-length string pair was strictly smaller than its edit distance. Then the edit distance could not have been the smallest number of single-character insertions, deletions, and substitutions to convert one string into the other, as the series of substitutions that resulted in the hamming distance would have converted one string into the other at an even smaller cost. Thus, the Hamming distance of an equal-length string pair cannot be strictly smaller than its edit distance.

11. Comprehensive GPU Scaling Results

We explore the scaling of Scrooge's throughput with GPU threads for W=64 and O=33. Supplementary Figure 11 is the extended version of Figure 8, showing all possible combinations of improvements. In particular, it shows combinations including and excluding Early Termination (ET). From Supplementary Figure 11, we observe that ET yields a small but consistent benefit when enabled. In contrast, enabling each of the memory improvements (i.e., SENE and DENT) yields a large throughput increase. Thus, we omit ET from Figure 8 for readability.

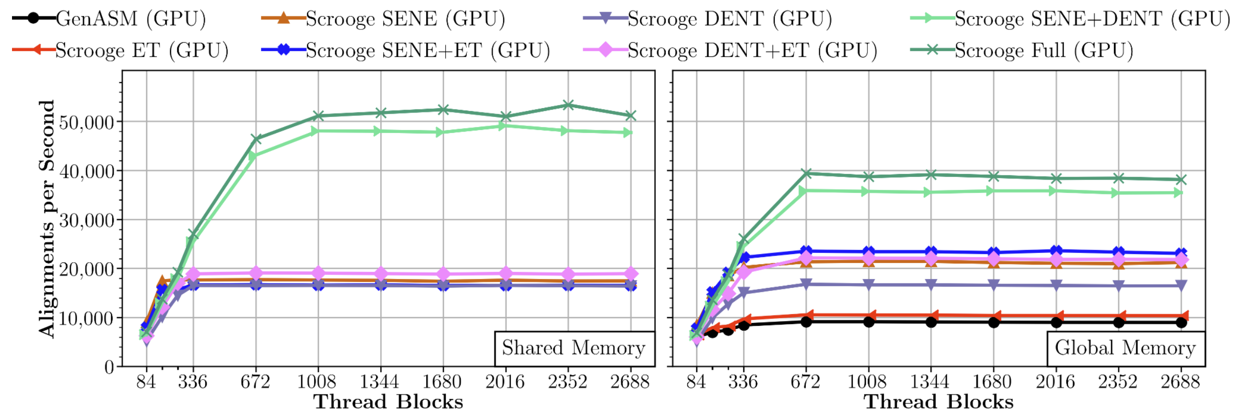

Suppl. Fig. 11: Scaling experiments of our GPU implementation with W=64, O=33, when the DP table placed in shared memory (left) and global memory (right).

12. Comprehensive CPU Scaling and Sensitivity to W Results

We explore the scaling of Scrooge's throughput with CPU threads for W=64 and O=33, and the sensitivity of throughput to the window size (W). Supplementary Figure 12 is the extended version of Figure 9, showing all possible combinations of improvements. In particular, it shows combinations including and excluding DENT. From Supplementary Figure 12, we observe that DENT yields small benefits at best (e.g., SENE+DENT vs.SENE), but can even lead to a slowdown (e.g., GenASM vs. DENT, SENE+ET vs. Full). In particular, for W=64 and O=33, the fastest configuration is SENE+ET doesn't use DENT. In contrast, enabling SENE and DENT yields consistent throughput increases. Thus, we omit DENT from Figure 9 for readability.

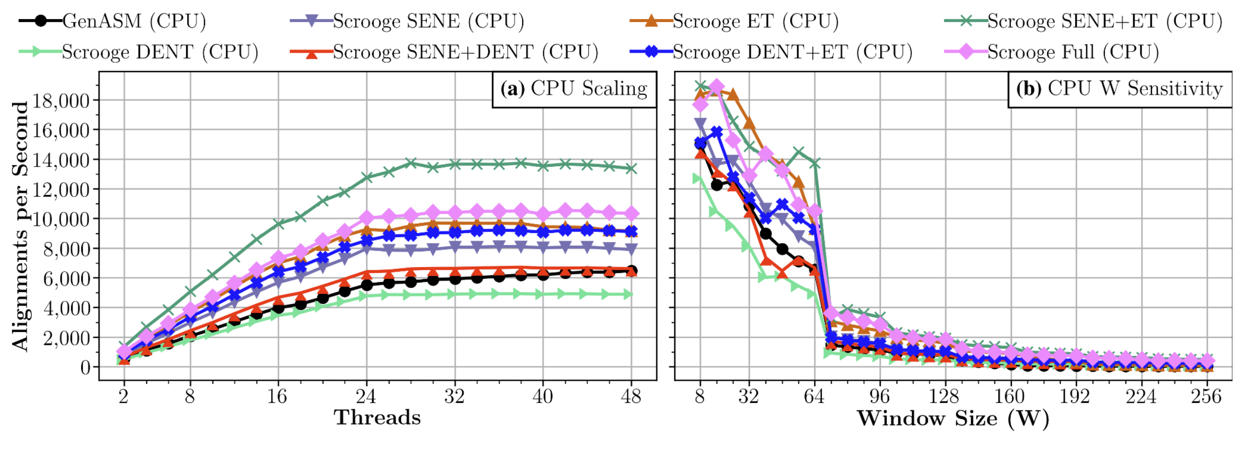

Suppl. Fig. 12: (a) Scaling and (b) sensitivity to window size of our CPU implementation.

13. Example of a “Difficult” Sequence Pair

We show in Section 3.6 that larger windowing parameters W and O generally improve alignment accuracy and that most sequence pairs are aligned optimally or near optimally. By manually inspecting a few worst-case (i.e., poorly aligned) sequence pairs, we observe that their apparent “difficulty” comes from small regions that (1) are relatively noisy and/or (2) contain repetitions of sequence pairs. We observe that if the windowing parameter W is increased to be larger than the noisy and/or repetitive region, these difficult sequence pairs *are* aligned correctly.

To further illustrate this observation, we give an example of an apparently “difficult” sequence pair from the long read groundtruth dataset in Supplementary Figure 13. Supplementary Figure 13 shows the alignment path produced by Edlib (i.e., a globally optimal edit distance alignment) and alignments produced by Scrooge for the windowing parameters (W=32, O=17) and (W=16, O=9). We observe that with (W=32, O=17), Scrooge aligns the sequence pair near optimally (the light green alignment by Scrooge almost completely covers the red alignment by Edlib). In contrast, Scrooge with (W=16, O=9) fails to find part of the optimal alignment early in the sequence pair (in the top left of Supplementary Figure 13) and never recovers for the remainder of the sequence pair.

Supplementary Figure 14 shows a zoomed-in view of where (W=16, O=9) first diverges from the optimal alignment. We make two key observations about this region of the sequence pair. First, we observe that it is particularly noisy, i.e., the optimal alignment contains 5 edits between reference characters 26 to 40 (an error rate of 33%). Second, we observe that it contains the highly repetitive strings “AGAGA” and “ACACACA” between reference characters 25 and 35. Scrooge with (W=16, O=9) fails to find the globally optimal alignment of these repetitive strings in favor of a more locally optimal alignment (i.e., it incurs only 2 edits until read character 31 instead of 3, which would be globally optimal). Scrooge with (W=32, O=17) also aligns “AGAGA” wrongly between reference characters 25 and 29 but has a sufficiently global optimal view to recover to the optimal alignment afterward. We conclude that the larger W parameter enables Scrooge with (W=32, O=17) to have a more globally optimal view of the noisy and repetitive region and hence produces a significantly better alignment.

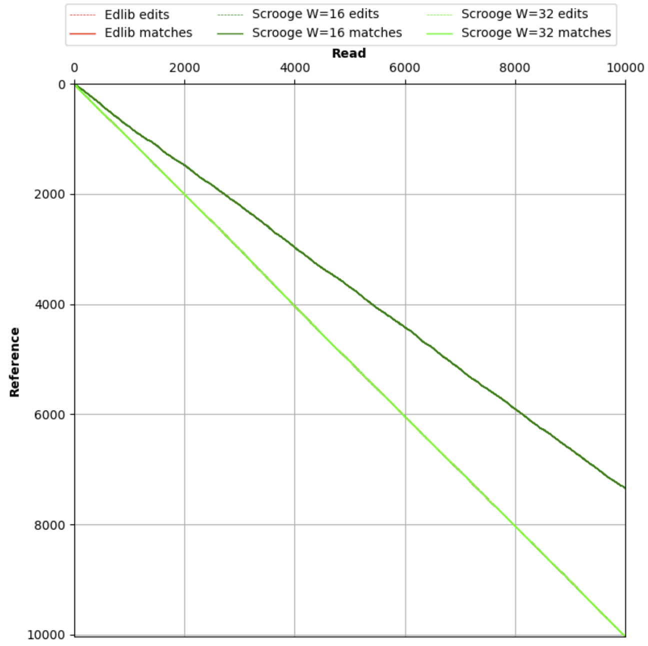

Suppl. Fig. 13: Alignment paths generated by Edlib and Scrooge with (W=32, O=17) and (W=16, O=9) for a single “difficult” sequence pair from the long read groundtruth dataset.

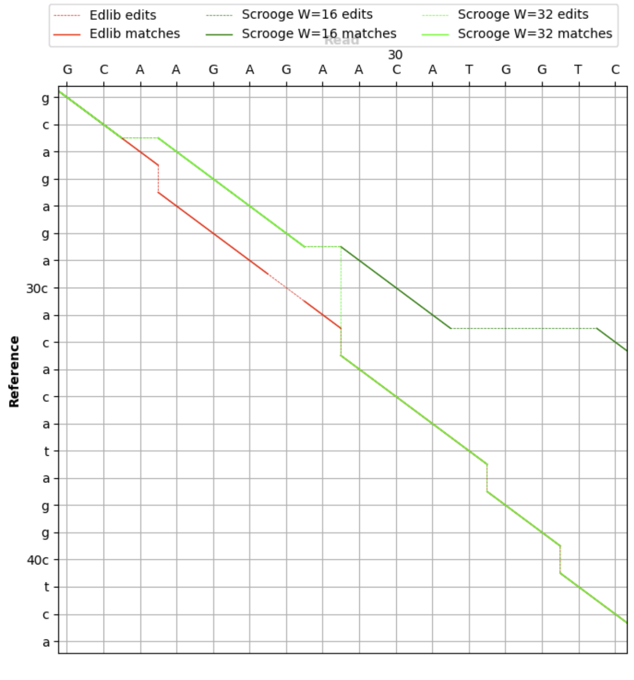

Suppl. Fig. 14: A zoomed-in view of the top left of Supplementary Figure 13, where Scrooge first diverges from the globally optimal alignment.

References

Hamming, R. W. (1950). Error Detecting and Error Correcting Codes. Bell Syst. Tech. J.

INTEL (2023). Intel 64 and IA-32 Architectures Optimization Reference Manual.

NVIDIA (2020). NVIDIA RTX A6000 Datasheet.

NVIDIA (2023). CUDA Programming Guide Release 12.0.

Ofenbeck, G. et al. (2014). Applying the Roofline Model. ISPASS.

Senol Cali, D. et al. (2020). GenASM: A High-Performance, Low-Power Approximate String Matching Acceleration Framework for Genome Sequence Analysis. MICRO.

Levenshtein, V. I. (1966). Binary Codes Capable of Correcting Deletions, Insertions, and Reversals. Soviet Physics Doklady.
